# Characterization of chromatin accessibility with a transposome hypersensitive sites sequencing (THS-seq) assay

**DOI:** 10.1186/s13059-016-0882-7

**Published:** 2016-02-04

**Authors:** Brandon Chin Sos, Ho-Lim Fung, Derek Rui Gao, Trina Faye Osothprarop, Amirali Kia, Molly Min He, Kun Zhang

**Affiliations:** Department of Bioengineering, University of California San Diego, 9500 Gilman Drive, La Jolla, CA USA; Biomedical Sciences Graduate Program, University of California San Diego, 9500 Gilman Drive, La Jolla, CA USA; Illumina Inc, 5200 Illumina Way, San Diego, CA USA

## Abstract

Chromatin accessibility captures *in vivo* protein-chromosome binding status, and is considered an informative proxy for protein-DNA interactions. DNase I and Tn5 transposase assays require thousands to millions of fresh cells for comprehensive chromatin mapping. Applying Tn5 tagmentation to hundreds of cells results in sparse chromatin maps. We present a transposome hypersensitive sites sequencing assay for highly sensitive characterization of chromatin accessibility. Linear amplification of accessible DNA ends with *in vitro* transcription, coupled with an engineered Tn5 super-mutant, demonstrates improved sensitivity on limited input materials, and accessibility of small regions near distal enhancers, compared with ATAC-seq.

**Electronic supplementary material:**

The online version of this article (doi:10.1186/s13059-016-0882-7) contains supplementary material, which is available to authorized users.

## Background

The accessibility of chromatin is a major determinant of gene regulation. The chromatin landscape defines the transcriptional regulatory networks that determine cellular identity and function as well as biological processes involved in differentiation, proliferation, development, and responses to the extracellular environment. Genome-wide chromatin accessibility assays were first developed utilizing cloning [1], then microarrays [2], and now existing assays such as DNase-seq [3], FAIRE-seq [4], and the recently developed ATAC-seq [5] methods have been shown to be remarkably powerful in defining the binding status of transcription factors, determining nucleosome occupancy, and constructing gene regulatory networks [6–14]. Most recently accessibility has been assayed at the single-cell level [15, 16]. However, the resulting data are rather sparse, such that single-cell analyses still need amalgamation of replicates for data analysis and to call significantly accessible regions. There is room for further improvement on the sensitivity of the tagmentation-based approach, which would facilitate routine profiling of accessible chromatin on a wide variety of samples with limited input.

The widely used DNase-Seq and ATAC-seq methods have several areas that can be improved upon to increase assay sensitivity. For DNase-Seq millions of cells are required for nuclei isolation, DNase I titration, downstream enzymatic reactions, and associated purification steps for DNA end polishing and adaptor ligations [3]. These inefficiencies were addressed by ATAC-seq and its usage of the Tn5 transposome system, which was originally developed for generating low input sequencing libraries [17, 18]. With this method, chromatin accessibility assays were demonstrated initially at the 50,000-cell level, and more recently with single cells. However, with the ATAC-seq method, we identified three aspects that can be potentially improved to increase the assay sensitivity. First, by design, the method uses PCR amplification immediately after Tn5 insertion, and only a fraction of the ‘tagmented’ molecules can be amplified and recovered, as not all inserted adaptor pairs are in the correct orientation or have the appropriate spacing to generate molecules of a size amenable to PCR amplification. Second, buffer conditions are critical for Tn5 activity, and critical components such as dimethylformamide (DMF) can be titrated for optimal Tn5 activity for assessing chromatin accessibility, as well as determining the optimal concoction of buffer components [19]. Third, the commercial EzTn5 transposase is a mutated version of the wild-type Tn5 enzyme that has high activity for random transposition [20, 21]. There is potential room to further engineer the enzyme to achieve a more efficient and specific insertion into open chromatin. Here we present a systematic effort to greatly increase the sensitivity of transposase-based DNA accessibility assays through optimization of all three aspects.

## Results and discussion

### THS-seq design and implementation

**Fig. 1:**
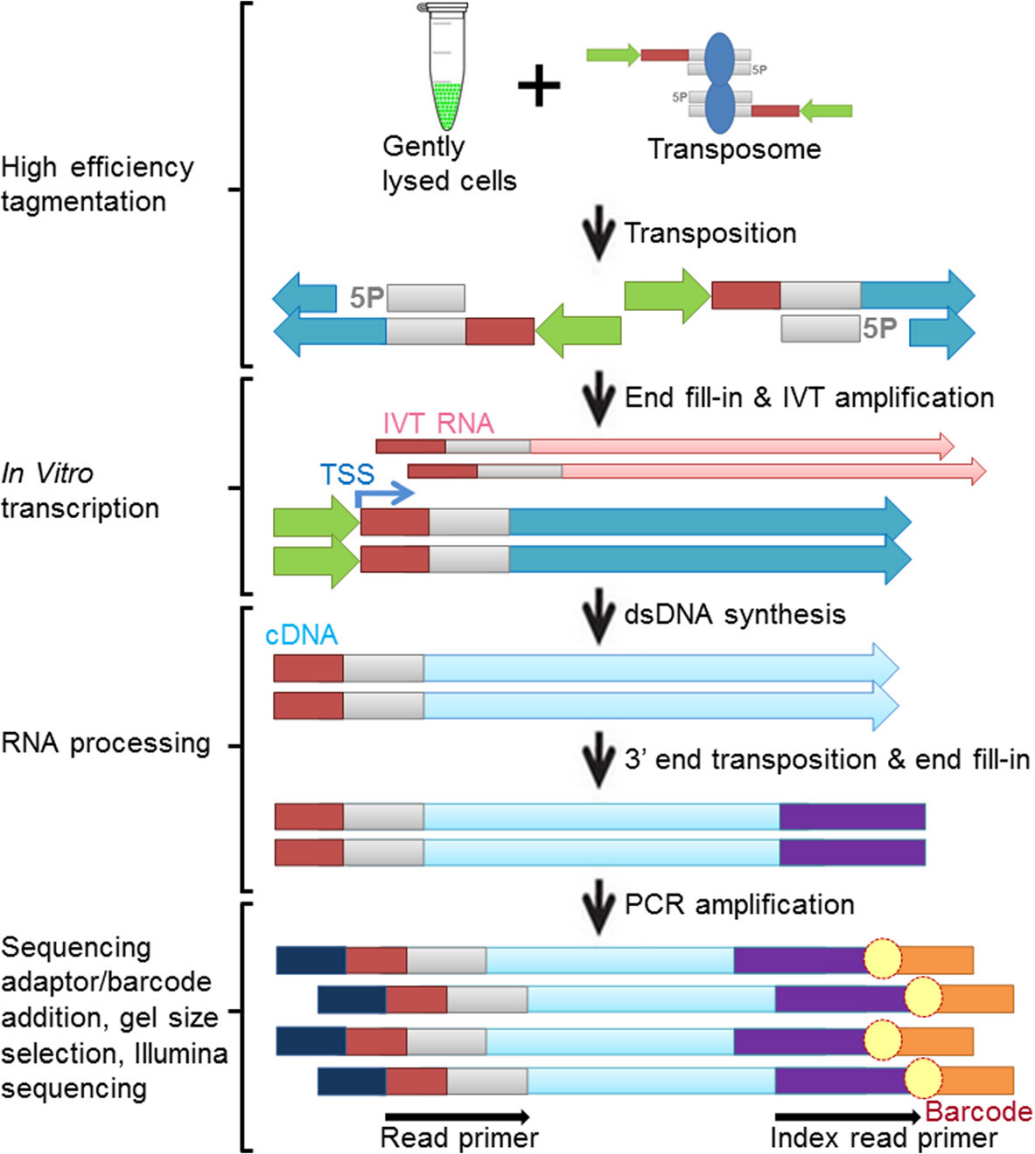
Schematic overview of THS-seq. High efficiency tagmentation is performed on gently lysed cells, followed by *in vitro* transcription, and custom RNA-seq to generate barcoded sequencing libraries. The following colors depict the following segments: light gray segments are Tn5 mosaic ends sequences, green segments are T7 promoter sequences, dark red segments are read primer sequences, dark blue segments are genomic DNA, light blue segments are cDNA sequences, purple segments are 3’ adaptor sequences, orange and navy blue segments are Illumina adaptor sequences, and yellow circles are barcodes

We hypothesized that the limited sensitivity of ATAC-seq is inherent in the method design, and is mainly due to three factors. First, the Tn5 transposome complex inserts adaptors in random orientation, such that only half of the molecules contain the adaptors in the orientation required for PCR amplification. Second, only approximately 1 % of the genome is accessible in typical cells, and the regions in which two adjacent transposition events are too far apart cannot be amplified by PCR. In fact, for this reason, the existing DNase-Seq method includes a fragmentation step to capture and sequence only the flanking sequences immediately adjacent to DNase I digestion sites, which effectively captures single-digestion events. Third, accessible regions small in length would have too few transposition events, and in conjunction with losing half of the molecules due to incorrect adaptor orientation, would not produce enough molecules to form a detectible peak above background levels of transposition events. Therefore, applying such a fragmentation strategy to small numbers of cells or single cells would result in low sensitivity, especially in the small accessible regions. We therefore developed the THS-seq method, which uses a customized Tn5 transposome system to attach a T7 promoter [22–25] to the end of every DNA molecule after *in vitro* transposition. The end sequences of the insertion sites were then linearly amplified with *in vitro* transcription by roughly 1,000-fold, regardless of the distance between two adjacent Tn5 insertion sites, and the resulting RNA molecules were then converted into sequencing libraries efficiently through seven enzymatic reactions (single-stranded cDNA synthesis, RNase H digestion, double-stranded cDNA synthesis, transposition, protease digestion, end fill-in, and PCR amplification) (Fig. 1). To this end we designed a custom transposon that, in addition to the mosaic end sequences for transposase binding, includes a T7 promoter sequence for *in vitro* transcription, and an adaptor sequence compatible with constructing Illumina sequencing libraries (Additional file 1: Figure S1). Additionally, to address the efficiency loss from incorrect adaptor orientations that is seen with PCR-based methods, the transposome complex dimer that consists of two Tn5 molecules and two transposons, where one Tn5 molecule is bound to one transposon, was designed and generated so that every single insertion yields usable ends regardless of the orientation of the transposon after insertion. Successful insertion of our customized transposon was confirmed by electrophoresis (Additional file 1: Figure S2). Next, all proteins were removed by treatment with guanidine hydrochloride, followed by end fill-in and linear *in vitro* transcription amplification by T7 RNA polymerase, which in theory has a lower bias compared with exponential PCR amplification. The resulting RNA molecules (Additional file 1: Figure S3a-d) were converted to double-stranded cDNA, followed by a second round of transposition to tag 3’-ends of double-stranded cDNA. Transposase was digested by protease to release DNA fragments and then to generate sequencing libraries, PCR amplification was performed that added sequence containing Illumina adaptors and a sample barcode (Additional file 1: Figure S4a-d). Multiple samples were tagged with sample-specific DNA barcodes, pooled, and size selected for Illumina sequencing. Finally, we replaced the standard EzTn5 transposase with a novel Tn5 super-mutant (Tn5059), which resulted from a semi-rational design and has a higher activity and less sequence-specific bias than EzTn5 on purified DNA. Concentration titrations were performed on Tn5059 for optimal activity for THS-seq with cellular input (Additional file 1: Figure S5a-d), as well as development of an optimized tagmentation reaction buffer that includes 16 % DMF (Additional file 1: Figure S5e, f).

### THS-seq validation

**Fig. 2:**
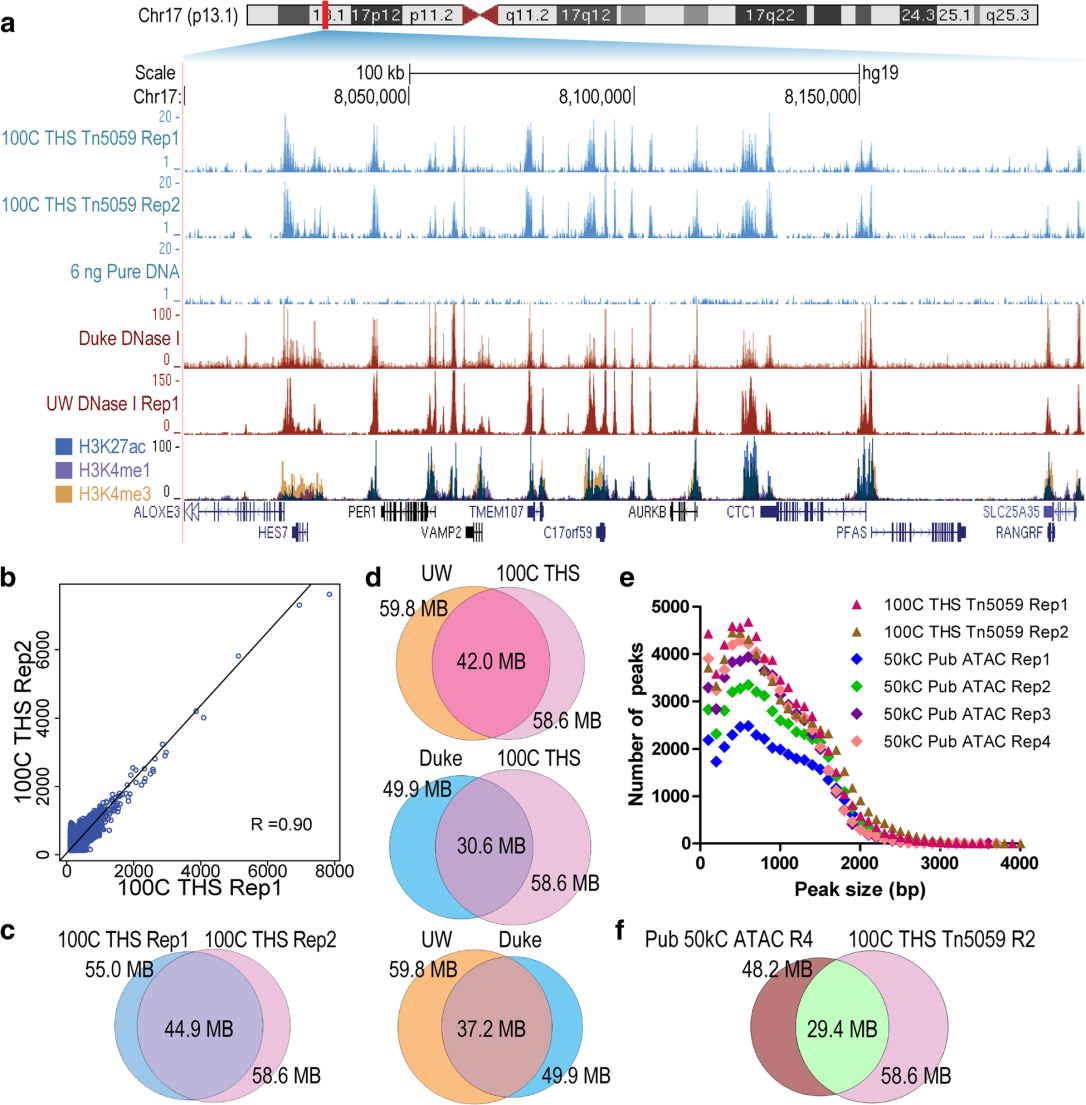
Validation of 100-cell THS-seq/Tn5059 data against ENCODE data and ATAC-seq data. **a** 200 kb view of accessible chromatin marks in GM12878 lymphoblastoid cells in 100-cell THS-seq/Tn5059 data, a purified DNA control, published ENCODE accessibility data from Duke and UW, and ENCODE histone modifications which are often found near regulatory elements and promoters. **b** Correlation of 100-cell THS-seq/Tn5059 replicate 1 data and 100-cell THS-seq/Tn5059 replicate 2 data. **c** Base pair overlap of 100-cell THS-seq/Tn5059 replicate 1 data and 100-cell THS-seq/Tn5059 replicate 2 data. **d** Base pair overlap between 100-cell THS-seq/Tn5059 replicate 2 data and ENCODE datasets, and ENCODE datasets base pair overlap among themselves. 100-cell THS-seq/Tn5059 replicate 2 data were used in ENCODE comparisons because they had the most base pairs in peaks called significant. **e** Peak size distributions between 100-cell THS-seq/Tn5059 datasets, and published 50,000-cell ATAC-seq datasets. **f** Base pair overlap between 100-cell THS-seq/Tn5059 and published 50,000-cell ATAC-seq replicate 4 data. Published 50,000-cell ATAC-seq replicate 4 data were used since they had the most base pairs called significant compared to the other published 50,000-cell ATAC-seq datasets

To validate THS-Seq, we applied the method to 100 GM12878 lymphoblastoid cells, and generated 71.5 and 83.4 million Illumina sequencing reads per replicate. After sequence alignment and clonal read removal, we obtained roughly 11 million uniquely mapped reads for each library. Each THS-seq unique read by design represents a unique transposon insertion event on a single chromosomal molecule. Therefore, approximately 110,000 unique transposition events per cell was captured in our dataset. We then performed peak calling with Dfilter [26], and observed consistency in the base pair overlap called between the two technical replicates (Fig. 2b, c). Chromatin accessibility profiles mimicked published ENCODE DNase I data of GM12878 (Fig. 2a). In comparison with ENCODE reference data there was 61 % base pair overlap with Duke data, and 70 % overlap with UW data from the same cell line [27], and this was comparable to base pair overlap between the ENCODE datasets from different labs when overlapped against each other (Fig. 2d, Additional file 1: Table S1). Additionally, base pair overlap between published 50,000-cell ATAC-seq data and 100-cell THS-seq/Tn5059 data was 61 %, which is comparable to ENCODE data base pair overlaps against themselves as well as the number of peaks called and peak size distributions from published 50,000-cell ATAC-seq data, indicating an approximately 500-fold improvement of sensitivity (Fig. 2d-f, Additional file 1: Table S2). This increase in sensitivity is accompanied by a 30 % increase in the total base pairs called significant by 100-cell THS-seq/Tn5059 over published 50,000-cell ATAC-seq data (Additional file 1: Table S3). In contrast, the control data generated from 6 ng of purified genomic DNA, where all proteins have been removed and DNA is completely open, yielded mostly non-specific peaks that have less than 1 % overlap with ENCODE datasets (Fig. 2a, Additional file 1: Table S1). Furthermore, an analysis by GREAT [28] shows accessible regions form a bimodal distribution of peaks around transcription start sites, suggesting that accessible regions largely lie over regulatory non-coding regions (Additional file 1: Figure S6a). Annotation of accessible regions identified enrichment of immune system and B lymphocyte biological processes, including interferon signaling, B cell receptor signaling pathway, and B cell homeostasis, which are known to be upregulated in EBV transformed lymphoblastoid cells [29–31] (Additional file 1: Figure S7a). There is a well-known limitation with ATAC-seq where 30–50 % of sequenced reads are clonal reads from the mitochondrial genome, however with 100-cell THS-seq/Tn5059, only 6 % of mapped reads came from the mitochondrial genome (Additional file 1: Figure S8a-c).

### Comparison of THS-seq and ATAC-seq with two transposases: Tn5059 and EzTn5

**Fig. 3:**
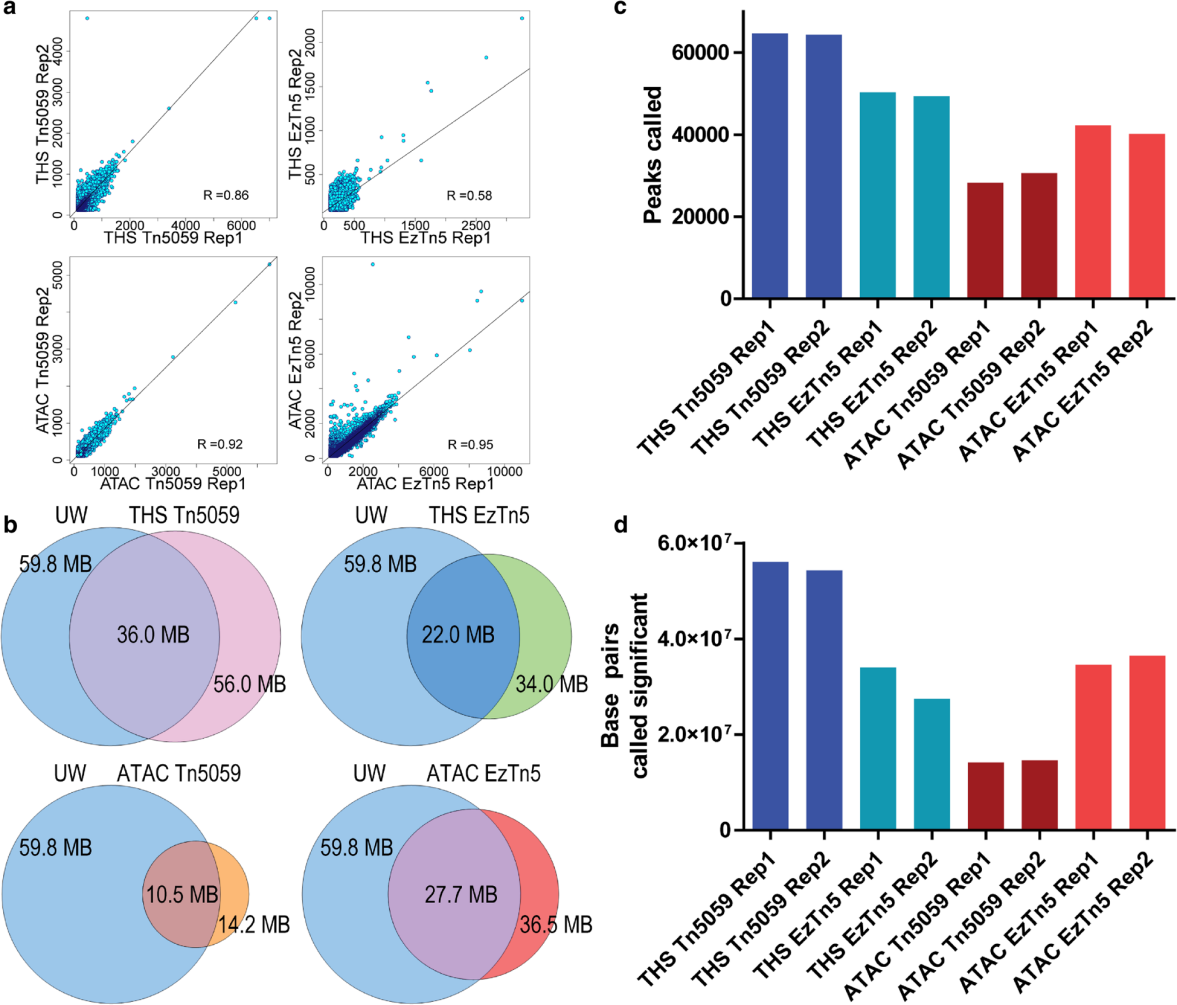
Comparison between THS-seq/Tn5059, THS-seq/EzTn5, ATAC-seq/Tn5059, and ATAC-seq/EzTn5 with 500 cells of input material. All datasets and replicates were down-sampled to 8,351,125 unique alignments before analysis. **a** Correlation between replicates for each experimental condition. **b** Base pair overlap of each experimental condition with UW data. The replicate with the most base pairs called significant was used in analysis and represented in each condition. UW data were chosen since they had the most base pairs called significant of the ENCODE datasets. **c** Total number of peaks called by Dfilter for each condition. **d** Total number of base pairs under peaks called significant by Dfilter

We next sought to identify the contributions to the improved sensitivity by individual components of THS-seq. More specifically, we asked whether a direct replacement of the transposase in ATAC-seq, which has fewer processing steps, would lead to a similar level of improvement, and whether Tn5059 led to a substantial improvement over EzTn5 with the THS-seq protocol. We used an input of 500 cells in order to directly compare with published 500-cell ATAC-seq data [5]. We performed ATAC-seq and THS-seq with EzTn5 and Tn5059, generating data for all four combinations in two replicates. To eliminate the effects of variable read depth among different datasets, we down-sampled all datasets to 8,351,125 uniquely aligned reads per sample, which matched the sample with the lowest number of unique alignments, and was adequate for generating accessibility data metrics (Additional file 1: Figure S9, Table S3, Table S4). Technical replicates consistently have high base pair overlap, peak overlap, and correlations with each other (Fig. 3a, Additional file 1: Figure S10a, S11a). 500-cell THS-seq/Tn5059 consistently performs the best by generating 61 % more significant peaks, calling 54 % more base pairs significant, having 45 % more peak overlap with ENCODE UW peaks, having 16 % more peak overlap with ENCODE Duke peaks, and having 30 % more base pairs overlapped with UW base pairs, when compared with 500-cell ATAC-seq/EzTn5 data, which is the next most comprehensive accessibility dataset (Fig. 3b-d, Additional file 1: Figure S10b, c, S11b, Table S3). Peak overlaps are comparable to ENCODE and published 50,000-cell ATAC-seq data overlap among themselves (Additional file 1: Figure S10e). Pairwise comparisons of peak overlap and base pair overlap between all four combinations show that THS-seq or ATAC-seq against each other, regardless of enzyme used, have the best overlap (Additional file 1: Figure S12, S13). The two most comprehensive datasets, 500-cell THS-seq/Tn5059 and 500-cell ATAC-seq/EzTn5, when overlapped against each other, have 62 % of the same peaks covered, and 50 % of the same base pairs covered, of the total from the 500-cell ATAC/EzTn5 dataset (Additional file 1: Figure S12, S13). Interestingly 500-cell ATAC-seq/EzTn5 has 7.7 % more base pair overlap with Duke data than 500-cell THS-seq/Tn5059, possibly because 500-cell ATAC-seq/EzTn5 data captures larger portions of peaks, where THS-seq instead captures individual peaks in the same region that are smaller (Additional file 1: Figure S10b, S11b). This can be explained by the increased sensitivity of THS-seq/Tn5059, which in this case would result in less base pair coverage and more overlapping peaks called. It could also be that the Duke data were generated differently due to protocol differences, and is capturing larger peaks [2, 3]. Compared with published 50,000-cell ATAC-seq data, 500-cell THS-seq/Tn5059 has 49 % more peak overlaps and 16 % more base pair overlaps than 500-cell ATAC-seq/EzTn5. 500-cell THS-seq/Tn5059 data also have more comparable peak overlap of 36,846 peaks to published 50,000-cell ATAC-seq data and Duke data peak overlap of 30,417 peaks, and 50,000-cell ATAC-seq and UW data peak overlap of 35,501 peaks. 500-cell ATAC-seq/EzTn5 peak overlap with 50,000-cell published ATAC-seq data are comparably less than ENCODE datasets overlap with 50,000-cell published ATAC-seq data, with 19 % less Duke data peak overlap, and 30 % less UW data overlap, with 24,759 peaks. (Additional file 1: Figure S10d, e, S11c). A higher fraction of overlapping peaks and base pairs between THS-seq and ENCODE reference data indicates that THS-seq not only has a higher sensitivity, but also a higher specificity, which could be due to either less sequence-specific bias or less off-target transposition or both.

THS-seq with EzTn5 provides an obvious improvement for all metrics over the published ATAC-seq data from 500 cells [5]. Compared with the 500-cell data we generated in this study using ATAC-seq/EzTn5 in our optimized protocol, THS-seq/EzTn5 identified 25 % more peaks that are smaller in size, such that 6.7 % less base pairs were covered (Fig. 3b-d, Additional file 1: Table S3). Unexpectedly, ATAC-seq/Tn5059 results in the least number of peaks called, base pairs called significant, and base pair overlap with ENCODE reference data (Fig. 3b-d, Additional file 1: Table S3). This is also reflected in the gene ontology analysis by GREAT [28], which reported ontology categories mostly not involved with immune system function for ATAC-seq/Tn5059 peaks (Additional file 1: Figure S7d). Furthermore, the data are reflected when visually inspecting the data tracts, where THS-seq/Tn5059 and ATAC-seq/EzTn5 have the most well defined peaks (Additional file 1: Figure S14).

**Fig. 4:**
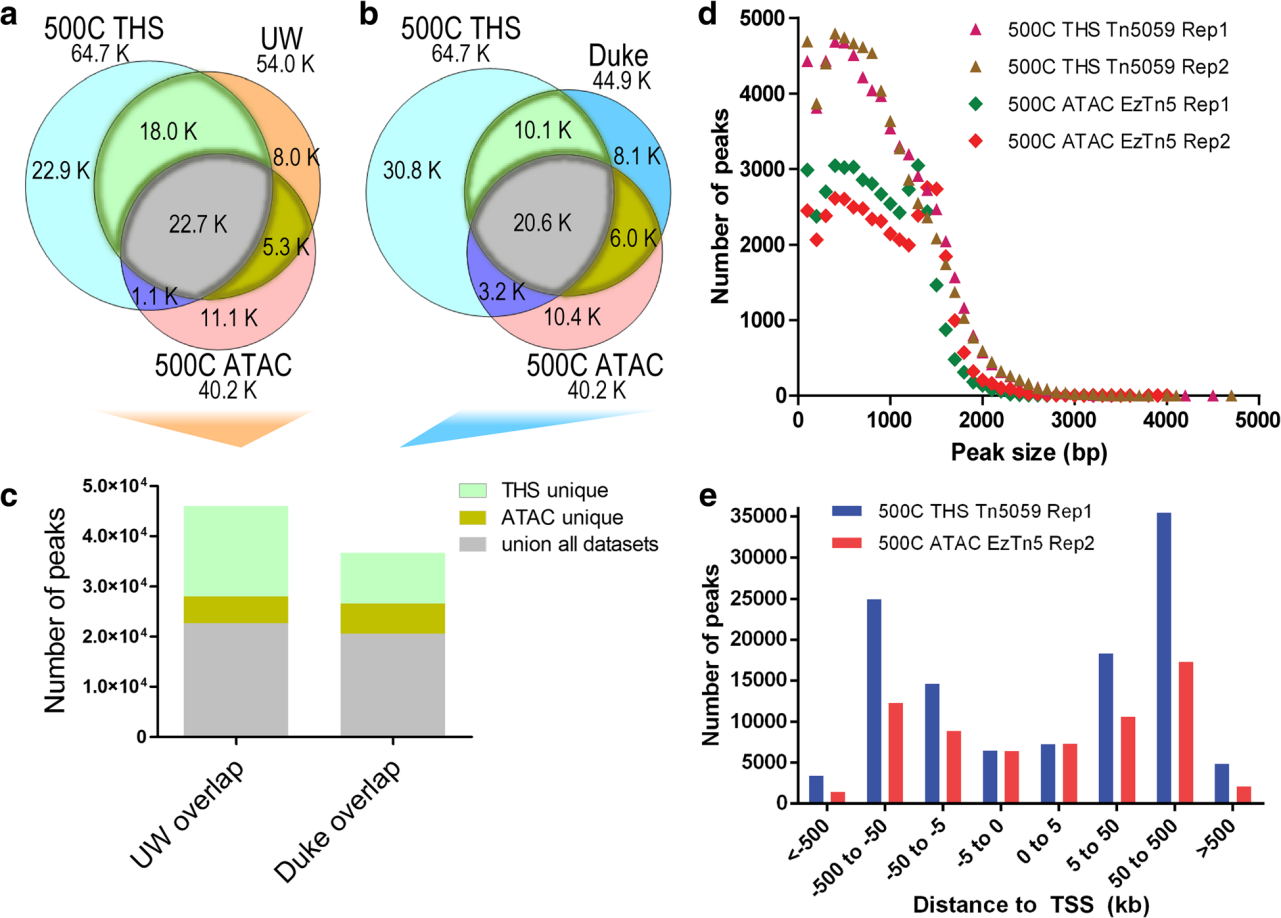
Comparison between the two most comprehensive datasets of 500-cell THS-seq/Tn5059 and 500-cell ATAC-seq/EzTn5. **a** Venn diagram depicting peak overlap between 500-cell THS-seq/Tn5059, 500-cell ATAC-seq/EzTn5, and ENCODE UW data. A peak is shared if 1 base pair or more overlaps with a peak in the dataset being compared to. **b** Venn diagram depicting peak overlap between 500-cell THS-seq/Tn5059, 500-cell ATAC-seq/EzTn5, and ENCODE Duke data. A peak is shared if 1 base pair or more overlaps with a peak in the dataset being compared to. **c** Representation of the number of peaks that are shared between all three datasets for UW data, peaks that are found by 500-cell THS-seq/Tn5059 data and ENCODE UW data and not 500-cell ATAC-seq/EzTn5 data, and peaks that are found by 500-cell ATAC-seq/EzTn5 data and ENCODE UW data and not 500-cell THS-seq/Tn5059 data. Also a representation of the number of peaks that are shared between all three datasets for Duke data, peak regions that are found by 500-cell THS-seq/Tn5059 data and ENCODE Duke data and not 500-cell ATAC-seq/EzTn5 data, and peaks that are found by 500-cell ATAC-seq/EzTn5 data and ENCODE Duke data and not 500-cell THS-seq/Tn5059 data. **d** Comparison of peak size distributions. **e** Peak distances from transcription start sites as determined by GREAT [28]

THS-seq and ATAC-seq, regardless of which enzyme was used, identified similar numbers of peaks, 5,098-7,310, within 5 kb of transcription start sites. However, THS-seq called 1.5 to two times more peaks in distal regions to transcription start sites, suggesting additional regulatory regions are being captured by THS-seq that are not being captured with ATAC-seq (Fig. 4e). This leads to gene ontology descriptions that appear more descriptive of GM12878 lymphoblastoids, such as the B cell receptor signaling pathway, and B cell homeostasis (Additional file 1: Figure S6, S7). Further inspection of these gene enrichments reveals genes where 100- and 500-cell THS-seq/Tn5059 was able to identify accessible regions, and 500-cell ATAC-seq/EzTn5 did not. Some notable examples include ABCA12, FCRL4, IFI44, PDGFD, and PHLDB2 (Additional file 1: Figure S15a-e). Genes including BCL2, CASP3, CD38, the interferon gene cluster and specifically IFNA2, and MAPK1 that have major roles in cancer and in immune system function were enriched by both 100- and 500-cell THS-seq/Tn5059 and 500-cell ATAC-seq/EzTn5, however 100- and 500-cell THS-seq/Tn5059 called two to six times as many peaks per gene region, and more accurately mirrored UW DNase-seq data from the same gene regions (Additional file 1: Figure S16a-e). Consistent with published results, ATAC-seq/EzTn5 mitochondrial reads account for more than 30 % of the total reads, and account for three times the percentage of mapped reads when compared to THS-seq. ATAC-seq/Tn5059 mitochondrial read percentages are similar to THS-seq, probably due to the difference of sequence preference between EzTn5 and Tn5059 (Additional file 1: Figure S8a, b).

We next sought to further characterize and directly compare the two most comprehensive datasets of 500-cell THS-seq/Tn5059 and 500-cell ATAC-seq/EzTn5. In order to determine the number of peaks that were found unique between ENCODE UW data and 500-cell THS-Seq/Tn5059 data, and not found by 500-cell ATAC-seq/EzTn5 data, a union dataset was made. Here we found 500-cell THS-seq/Tn5059, 500-cell ATAC-seq/EzTn5, and UW had 22,700 peaks in common, while 500-cell THS-seq/Tn5059 data had 240 % more uniquely identified peaks that overlap with UW data than 500-cell ATAC-seq/EzTn5 data (Fig. 4a-c). The same analysis was performed with Duke data, though 9.3 % less peaks were found in common between all datasets, and 500-cell THS-seq/Tn5059 had 68 % more uniquely identified peaks that overlap with Duke data than 500-cell ATAC-seq/EzTn5 data (Fig. 4b, c). Next, we examined the distribution of peak sizes for each dataset, and found peaks in the smaller size range of 100-1,200 base pairs and larger size range of 1,300-3,000 base pairs, relative to 500-cell ATAC-seq/EzTn5, are gained with 500-cell THS-seq/Tn5059 (Fig. 4d, Additional file 1: Table S2). These peaks are present in more proximal and distal regions from the transcription start site, suggesting involvement in regulatory regions (Fig. 4e). The best published 50,000-cell ATAC-seq datasets approach the capture rate of smaller peaks by THS-seq/Tn5059, suggesting higher sensitivity Tn5 based methods are able to capture these peaks (Additional file 1: Figure S17f, g).

**Fig. 5:**
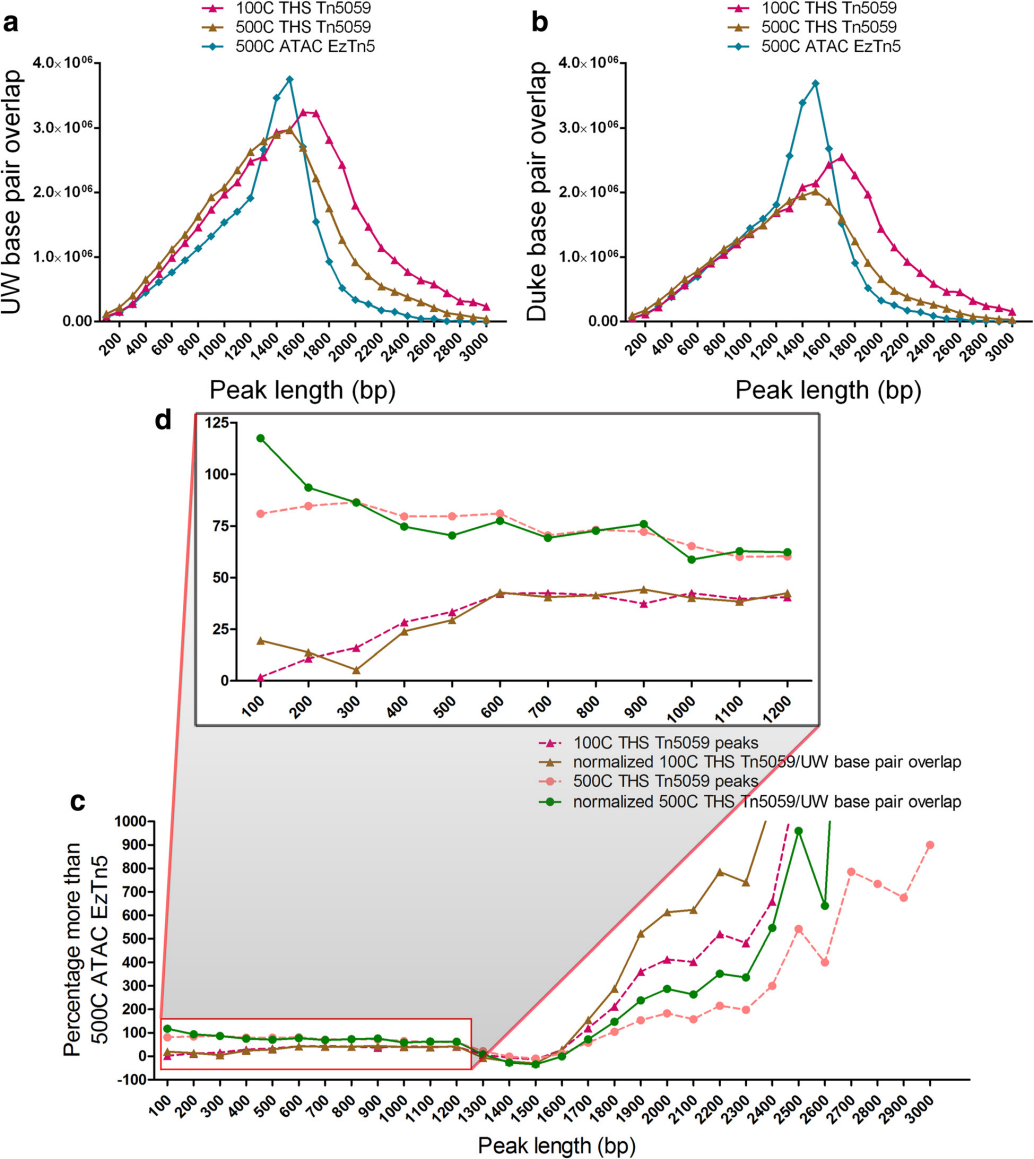
Validation of peaks based on peak length. **a** Base pair overlap with ENCODE UW data based on peak lengths for 100-cell and 500-cell THS-seq/Tn5059 data, and 500-cell ATAC-seq/EzTn5 data. **b** Base pair overlap with ENCODE Duke data based on peak lengths for 100-cell and 500-cell THS-seq/Tn5059 data, and 500-cell ATAC-seq/EzTn5 data. **c** The percentage more peaks found in 100-cell and 500-cell THS-seq/Tn5059 than in 500-cell ATAC-seq/EzTn5 data, and the percentage more normalized 100-cell and 500-cell THS-seq/Tn5059 and UW base pair overlap than in 500-cell ATAC-seq/EzTn5. Normalizing was performed using the global base pair overlap values for each ENCODE dataset. **d** Zoom in on graph (**c**) showing the peak lengths between 100-1,200 base pairs

We next sought to confirm that these extra peaks 100-1,200 base pairs in length are not artifacts generated by THS-seq, but instead have biological significance. A possibility is that these smaller peaks overlap in portions of larger peaks in 500-cell ATAC-seq/EzTn5 data, and thus are being counted as parts of single large peaks, or could be a unique set of called peaks. To investigate this, we examined the total base pair overlap with ENCODE Duke and UW DNase-Seq data for each individual peak length. Since 75 % and 125 % more peaks in the size range of 100-1,200 base pairs and 1,700-3,000 base pairs, respectively, are identified by 500-cell THS-seq/Tn5059 (Fig. 4d), we would expect a marked increase in the number of significant base pairs that overlap with ENCODE data at these peak lengths. Indeed, with ENCODE UW data as the reference we observed an increase in overlapping base pairs in peaks 100-1,200 base pairs and 1,700-3,000 base pairs (Fig. 5a), suggesting the additional small peaks detected by THS-seq are also present in ENCOCE UW data. However, when examining the Duke data, we found no difference among all three datasets in overlapping base pairs with peaks 100-1,200 base pairs, but more base pair overlap with THS-seq data and Duke data in the range of 1,700-3,000 base pairs (Fig 5b). Therefore, there is a difference in the peaks called by the two ENCODE datasets (Duke vs. UW) that needs to be taken into account during analysis. Interestingly, the spike in base pair overlap seen from 1,300-1,600 base pairs in 500-cell ATAC-seq/EzTn5 data is due to both ATAC-seq/EzTn5 and ENCODE Duke and UW data better capturing peaks in the 1,300-1,600 base pair size range (Fig. 5a, b, Additional file 1: Figure S17a, b, e). Moreover, to further validate the extra peaks found with THS-seq/Tn5059, we examined the percentage more identified peaks than 500-cell ATAC-seq/EzTn5, and the normalized THS-seq/Tn5059 and ENCODE UW overlap. We would expect that when we see 60–80 % more peaks called in 500-cell THS-seq/Tn5059 than ATAC-seq/EzTn5, we would see 40 % less base pairs overlap, which is what is actually seen for peaks 100-1,200 base pairs in length (Additional file 1: Figure S18a, b). Global ENCODE UW overlap is 60 % for 500-cell THS-seq/Tn5059 data, and 70 % for 100-cell THS-seq/Tn5059 data. Normalizing THS-seq/Tn5059 and UW base pair overlap with those values shows the extra peaks called by THS-seq/Tn5059 in the 100-1,200 base pair size range overlap at the expected percentages (Fig. 5c, d). Additionally, there are more normalized base pair overlaps with 100 base pair peaks and 1,700-3,000 base pair peaks in 100- and 500-cell THS-seq/Tn5059, suggesting these peaks have higher base pair overlap then the average global ENCODE UW overlap percentage (Fig. 5c, d, Additional file 1: Figure S18a). This further suggests these THS-seq peaks represent true accessible regions, not artifacts.

Interestingly the same results are not reflected with ENCODE Duke data when examining normalized THS-seq/Tn5059 and ENCODE Duke base pair overlaps, when normalized to ENCODE Duke global overlap of 61 % for 100-cell data, and 49 % for 500-cell data (Additional file 1: Figure S18c-f). The normalized overlap percentage is greatly below the percentage of peaks called, for peaks 100-1,200 base pairs in length, suggesting these peaks are not present in ENCODE Duke data. The exception is with normalized 500-cell THS-seq/Tn5059 ENCODE Duke base pair overlaps, where peaks 100-300 base pairs in length are above and closely match the percentage more peaks, suggesting these additional peaks are also present in ENCODE Duke data (Additional file 1: Figure S18e, f). Interestingly there are 70 % more normalized THS-seq/Tn5059 and ENCODE Duke base pair overlaps with 100 base pair peaks, and more normalized base pair overlaps with peaks greater than 1,700 base pairs, suggesting these peaks have higher base pair overlap with ENCODE Duke data than the average global overlap percentage (Additional file 1: Figure S18e, f). Overall, we see some differences between THS-seq data and ENCODE Duke data that are not seen between THS-seq and ENCODE UW data, which could be due to factors such as protocol differences, sample preparation, or stochastic differences in the GM12878 cells assayed.

**Fig. 6:**
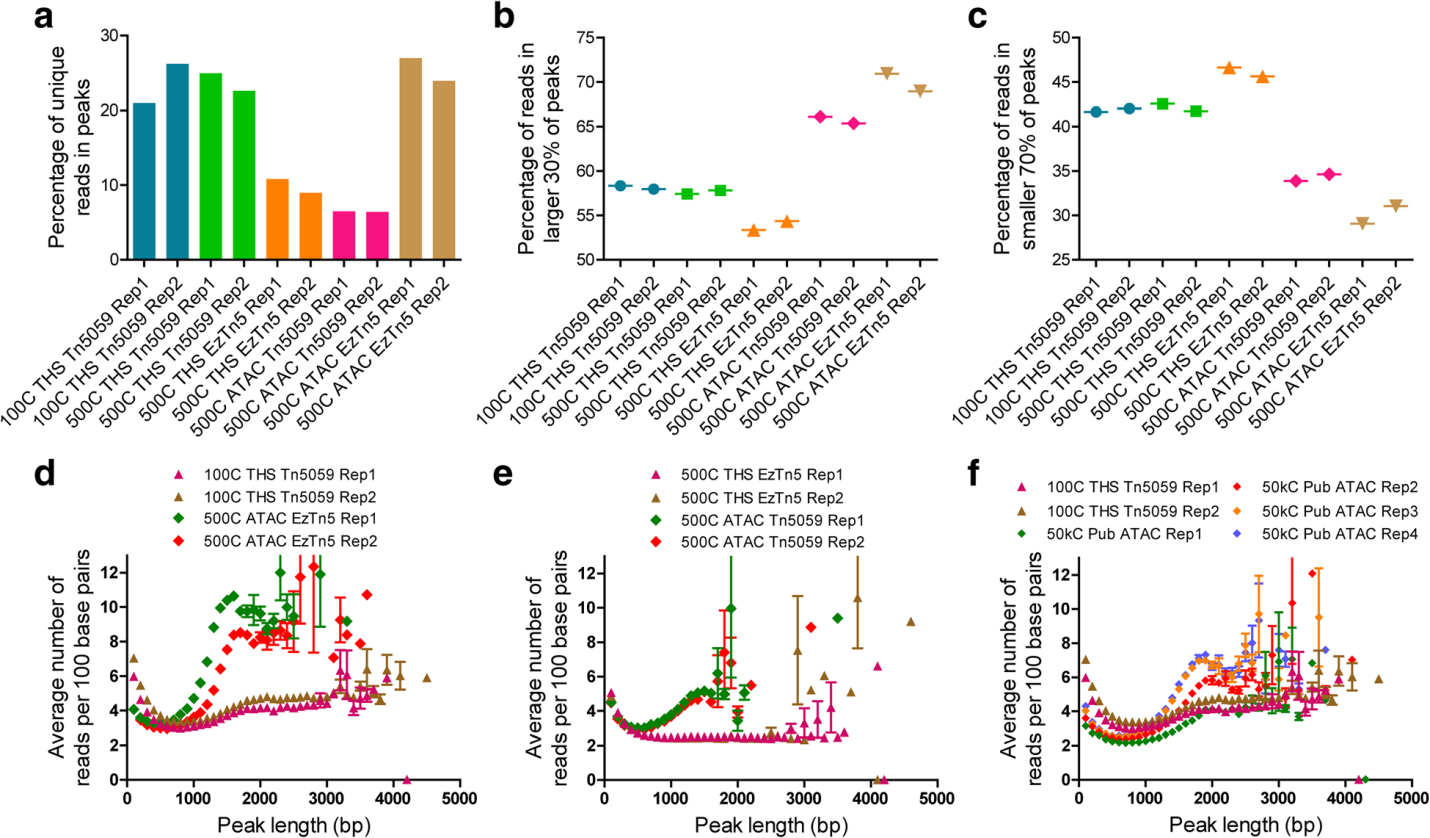
THS-seq and ATAC-seq peak capture preferences and biases. All datasets and replicates were down-sampled to 8,351,125 unique alignments before analysis. **a** Total percentage of unique alignments in peaks out of the 8,351,125 unique alignments for each dataset. **b** The percentage of alignments that are in the larger 30 % of peaks called significant for each individual sample and replicate. **c** The percentage of alignments that are in the smaller 70 % of peaks called significant for each individual sample and replicate. **d**-**f** For all peaks in each individual dataset, the normalized number of alignments in each peak length, with peak lengths in increments of 100 base pairs, represented by mean ± SEM in (**d**) 100-cell THS-seq/Tn5059 data and 500-cell ATAC-seq/EzTn5 data, (**e**) 500-cell THS-seq/EzTn5 data and 500-cell ATAC-seq/Tn5059 data, and (**f**) 100-cell THS-seq/Tn5059 data and published 50,000-cell ATAC-seq data. Some data points were excluded from the graphs because values were beyond the axis, and the number of data points excluded for each graph is: (**d**) 13, (**e**) 16, and (**f**) 8

We next investigated further the open chromatin regions, or peaks, called with the four combinations. Both THS-seq/Tn5059 and ATAC-seq/EzTn5 have high percentages of alignments in peaks, with 25 % and 27 %, respectively. Replacement of EzTn5 with Tn5059 in THS-seq results in 2.0 to 2.8 times more unique alignments in peaks (Fig. 6a). From this it can be conjectured that Tn5059 has higher preference for areas of open chromatin and/or EzTn5 has a higher background sporadic transposition activity in more compact chromatin, which explains how THS-seq with EzTn5 has significantly more background noise. For ATAC-seq with EzTn5, this is actually beneficial when considering the areas directly adjacent to an accessible region are semi-accessible. EzTn5 would insert more into regions around an open region than would Tn5059 and would generate more PCR viable fragments. This then semi-compensates for the 50 % sample loss from using ATAC-seq. Moreover, this explains how ATAC-seq with Tn5059 has the least number of unique alignments in peaks. Coupled with Tn5059’s more selective insertion properties, ATAC-seq’s inherent 50 % sample loss upon transposition dilutes the number of alignments in any peaks by at least 50 %, and hinders capture of any smaller regions of open chromatin. This is reflected in peak size distributions, where both ATAC-seq/Tn5059 and THS-seq/EzTn5, have 49 % and 30 % of peaks, respectively, in the size range of 100-300 base pairs long, which is 2 to 3 times the number of peaks found in that size range for THS-seq/Tn5059 or ATAC-seq/EzTn5 (Additional file 1: Figure S17c, d, Figure S19f, g, Table S2). We hypothesized that higher background transposition events with THS-seq/EzTn5, and sample loss with ATAC-seq/Tn5059 raised the thresholds for calling significant peaks, thus resulting in only the most significant portions of peaks being called significant and leading to smaller peak sizes. Further evidence reinforcing this interpretation is seen when examining the number of unique alignments within significantly called peaks, where THS-seq/EzTn5 and ATAC-seq/Tn5059 have the lowest with 11 % and 6.4 % of unique alignments in peaks, respectively (Fig. 6a). Here, the percentage of alignments in peaks is indicative of peak capture efficiency.

We next characterized peak capture bias in ATAC-seq, where preferentially peaks of longer length were captured, and peaks of shorter length were lost. ATAC-seq/EzTn5 had 20 % more alignments in the larger 30 % of peaks than did THS-seq/Tn5059, and concomitantly 27 % less alignments in the smaller 70 % of peaks, respectively (Fig. 6b, c). Moreover, this is further illustrated as THS-seq/Tn5059 with both 100 and 500 cells of input material has an even distribution of normalized alignments over all peak lengths when compared to ATAC-seq/EzTn5, where normalized alignments increase as peak length increases beginning around a peak length of 1,300 base pairs (Fig. 6d, Additional file 1: Figure S19a, b, d, e, h). The trend of more alignments in larger peaks is method consistent irrespective of enzyme used, and is seen in published 50,000-cell ATAC-seq data, though less so with published 500-cell ATAC-seq data due to sparse peak capture (Fig. 6b-f, Additional file 1: Figure S19a-c, Figure S20a, b). We hypothesize the reason for THS-seq’s more uniform capture of accessible regions is due to method design, usage of Tn5059, and is a result of the length of amplified molecules. The importance of fragment length in determining the type of biological data captured has been demonstrated with DNase-FLASH [32], and with THS-seq the majority of fragments generated from *in vitro* transcription were 400-2,000+ base pairs, while the majority of fragments from PCR amplification with ATAC-seq were 200-800 base pairs (Additional file 1: Figure S3a, c). Additionally, with THS-seq, the downstream transposition step for addition of 3’ adaptors favors longer double-stranded cDNA fragments. With longer fragment sizes and the hypothesis Tn5059 has higher preference for open chromatin, it is probable that THS-seq/Tn5059 is losing sample due to insertions being too close to each other in accessible regions of longer length, which does not happen with ATAC-seq since PCR amplification favors smaller fragment sizes. This would lead to ATAC-seq’s preference for capturing larger accessible regions. However, this does not affect THS-seq peak capture, as this would only occur in large regions of accessible chromatin. It is possible to further modify the RNA processing method in THS-seq to capture shorter fragments. Taken together, these data demonstrated that THS-seq/Tn5059 is feasible and improves upon current Tn5-based methods for measuring chromatin accessibility with limited inputs. This is achieved by reducing peak capture bias introduced by ATAC-seq, while providing data from 100 cells comparable to DNase-seq on millions of cells, or ATAC-seq on 50,000 cells.

### ATAC-seq improvements

Through optimizations to THS-seq, we inadvertently have made substantial improvements to ATAC-seq/EzTn5. Published 500-cell ATAC-seq data had on average 69 % less peaks called, 70 % less total base pairs called significant, and 61–64 % less ENCODE base pair overlap compared with published 50,000-cell ATAC-seq data (Additional file 1: Figure S20a-d, Table S3). Upon examination of peak size distributions, published 500-cell ATAC-seq data replicate 2 had 78 % of all peaks in the 100-300 base pair range, thus indicating high background noise, while replicate 1 had 17,331 peaks called (Additional file 1: Figure S17h, Table S2, Table S3). When we performed ATAC-seq/EzTn5, two changes were made to the published protocol. First, the tagmentation reaction contained 16 % DMF as final concentration in reaction, and second, after tagmentation protease digestion was performed on tagmented DNA and any cellular proteins in solution. These changes were able to generate data from 500 cells that had 131 % more peaks called, 169 % more base pairs called significant, and 141 % more ENCODE data base pair overlap than published 500-cell ATAC-seq data. This in turn generates data of higher quality, identifying more discernable chromatin accessibility peaks than published 500-cell ATAC-seq data (Additional file 1: Figure S17e, g, h, Figure S21). For 500 cells, our optimized ATAC-seq protocol with EzTn5 produced data at a quality approaching that of published 50,000-cell ATAC-seq data, albeit with 28 % less peaks called, 20 % less base pairs called significant, and 5–14 % less ENCODE base pair overlap (Additional file 1: Figure S20a-d, Table S3). Together these data illustrate how minor optimizations in protocol can substantially improve ATAC-seq for assessing chromatin accessibility, however still to a lesser degree than THS-seq/Tn5059.

## Discussion

In summary, THS-seq achieves a high sensitivity and specificity in detecting accessible chromatin by efficient *in vitro* transposition with a customized transposon, an optimized tagmentation buffer and an engineered Tn5 super-mutant, as well as linear RNA amplification of tagmented DNA molecules. We have validated THS-seq/Tn5059 against ENCODE data, and have shown it captures many peaks ATAC-seq/EzTn5 does not, including a large number of smaller peaks with probable roles in more proximal and distal regulatory regions to transcription start sites. The superior performance of THS-seq with Tn5059 over ATAC-seq with either enzymes, or THS-seq with EzTn5, represents an interesting case where the contributions of individual components are not additive, since changing the method from ATAC-seq to THS-seq while keeping the original EzTn5 provides only a subtle improvement, whereas replacing EzTn5 with Tn5059 is detrimental to ATAC-seq. Previously uncharacterized ATAC-seq bias, where peaks of longer length are preferentially captured, and peaks of shorter length are lost, reduces the comprehensiveness of ATAC-seq generated chromatin accessibility maps. Furthermore, as an unexpected result of this study, an optimized ATAC-seq protocol, is widely applicable to many current and future studies on small numbers of cells or single cells [16]. Although the lowest amount of input material used in this study was 100 cells, THS-seq is compatible with the high throughput combinatorial cell barcoding strategy [15] and when applied to single cells should lead to substantially denser maps of single-cell chromatin accessibility. With THS-seq, we captured >110,000 insertion events per cell. This compares favorably with what was recently reported with combinatorial ATAC-seq, which had a median of 1,685 reads per cell, and scATAC-seq using the C1 device to isolate single cells, with an average of 73,000 fragments per cell of those cells that pass filter [15, 16]. Adaptation of THS-seq to the combinatorial barcoding scheme will facilitate a much more comprehensive characterization of accessible chromatin landscapes in tens of thousands of single cells.

## Online methods

### Cell culture

GM12878 lymphoblastoid cells were obtained from Coriell Cell Repositories and maintained in RPMI 1640 with 1 % Penstrip and 10 % FBS. Cells were kept in suspension at 37 °C and were harvested when suspensions reached optimal density. Cell numbers were quantitated with a hemocytometer (Biorad), pelleted at 250 × g for 4 min and washed twice with a volume of PBS sufficient to obtain approximately 1,000,000 cells per mL in approximately 1 mL of PBS, and diluted as needed in PBS for experiments. All biological replicates were performed with material from the same cell culture flask, with independent running of the assays. When cells were diluted to 100,000 cells per mL they were quantitated again on the hemocytometer.

### Transposome generation

The transposon consisted of two DNA fragments synthesized by IDT, T7tspn-top2 (PAGE purified) and T7tspn-bot (Additional file 1: Table S5). These two fragments were incubated together to form annealed transposon at a concentration of 30 μM per oligo in Qiagen EB buffer for 2 min at 95 °C, and cooled to room temperature at 0.1 °C/s. Transposome generation by addition of transposase and annealed transposon, using either EzTn5 or Tn5059 was performed at Illumina. Prepared transposome was stored at −20 °C.

### Cell lysis, tagmentation, and DNA fragment processing

Cells were concentrated and aliquoted so the total number of cells was present in 1 μL optimally. Cells were lysed in 1× lysis buffer in reaction (10× concentration: 100 mM Tris-HCl pH 7.5, 100 mM NaCl, 30 mM MgCl2, 1 % NP40), at room temperature for 3–5 min by adding 1.0 μL of 2× lysis buffer to the cell mixture. Next, the transposition reaction was carried out at 37 °C for 30 min by adding 1.0 μL tagmentation buffer (5× concentration: 165 mM Tris-OAc pH 7.8, 330 mM K-OAc, 50 mM Mg-OAc, 80 % dimethylformamide), 1.0 μL nuclease-free water, and 1.0 μL of prepared transposomes (added last) to lysed cells for a total reaction volume of 5 μL. EzTn5 transposomes were added at a final concentration in reaction of 0.5 μM, and Tn5059 transposomes at a final concentration in reaction of 0.7 μM. After transposition, reactions were brought to 15 μL with nuclease-free water, and 15 μL of 8 M Guanidine HCl was added to degrade chromatin associated proteins and transposase. Next, reactions were purified with 1.8× (54 μL) SPRI beads. After the final wash with 80 % ethanol and brief air drying of SPRI beads, 9.6 μL of nuclease-free water was added. Importantly, the purified DNA products were not eluted off beads, and DNA products, nuclease-free water, and beads remained together in solution. Gap fill-in was performed by addition of 2.4 μL of 5X Taq polymerase solution (NEB), mixed thoroughly, and incubated at 72 °C for 3 min.

### *In vitro* transcription

DNA was used directly, in the same tube, and remaining in solution with SPRI beads after gap filling for *in vitro* transcription. Standard protocol with the MAXIscript® T7 Kit (Ambion) was followed. The 10× transcription buffer was kept at room temperature, and if crystals were present, heated slightly at 37 °C until crystals dissolved and returned to room temperature. Also as per manufacturer’s recommendation, after briefly thawing, ribonucleotides were stored on ice. Reactions were assembled at room temperature, with addition of 2 μL 10× transcription buffer, 1 μL 10 mM ATP, 1 μL 10 mM CTP, 1 μL mM GTP, 1 μL 10 mM UTP, and 2 μL T7 enzyme mix to 12 μL of reaction products. Reactions were incubated at 37 °C for 16-19 h. After *in vitro* transcription, RNA was purified with Zymo RNA Clean and Concentrator and eluted with 10 μL nuclease-free water. For purification, reactions were brought to 40 μL with nuclease-free water before starting the protocol, and whole reactions including SPRI beads in solution were applied to the RNA purification column. Reaction products were visualized and quantitated with a UV-transilluminator after running 1 μL of IVT product on a 6 % Tris-Borate-Urea (TBU) gel for 20-25 min at 250 V and staining with SYBR gold. For each sample the region from 250 base pairs to 2,000 base pairs was used for RNA quantitation and determination of the volumes of product needed for input into RNA-seq processing, since that portion of the gel is amplified RNA product. Fifty nanograms of RNA were used for input into RNA-seq processing.

### RNA-seq (custom transposon mediated fragmentation)

For first stranded cDNA synthesis the standard Clontech SMART MMLV reverse transcriptase protocol was followed. 2.5 μL of 20 μM random hexamers were first added to 50 ng RNA for each sample and incubated at 70 °C for 3 min then cooled immediately on ice. MMLV reverse transcription mastermix was added to make a 20 μL total reaction volume, followed by incubation at room temperature for 10 min, and then 42 °C for 60 min, and inactivated at 70 °C for 10 min. To remove RNA in DNA/RNA hybrids, samples were incubated for 20 min with 0.5 Units/μL RNase H at 37 °C. DNA templates were primed with 2.5 μL 20 μM sss_NPA_prmr (Additional file 1: Table S5) and incubated for 2 min at 65 °C then cooled immediately on ice. For second stranded synthesis 5.88 μL of 5× Taq PCR master mix (NEB) was incubated with samples at 72 °C for 8 min. Next, 45 μL nuclease-free water was added to reactions, and then double-stranded DNA was purified with SPRI beads at 1:1.8× ratio (135 μL SPRI beads), eluted with 20 μL nuclease-free water and then concentrated to approximately 4 μL in a Eppendorf™ Vacufuge™ Concentrator, set to 30 °C and spun for approximately 15–18 min. Fragmentation and 3’ end tagging of double-stranded cDNA was simultaneously performed by adding 1 μL tagmentation buffer, and 1 μL loaded custom 0.1 μM final in reaction EzTn5 transposomes (ME_BOT, ME_TOP (Additional file 1: Table S5)) to the solution and then incubating at 55 °C for 6 min, followed by cooling on ice. The same tagmentation buffer that was used in the transposition reaction for tagging open chromatin was used for this reaction. 0.14 μM final in reaction Tn5059 can also be used in place of EzTn5 and would provide better read diversity, however for this experiment we did not have Tn5059 with ME_BOT and ME_TOP available. Also, this transposome complex is prepared the same way as described in transposome generation above, however with different oligos as specified. Next, samples were incubated with 0.1 AU Qiagen protease at 50 °C for 10 min, then 70 °C for 20 min. Gap fill-in was performed by addition of 6 μL of 2× Taq PCR master mix (NEB), and incubated at 72 °C for 3 min. To the sample, 9.0 μL of 2× Taq was added with 2 μL of 10 μM of 5’ PCR primer, 2 μL of 10 μM Index barcode primer (for each individual unique sample) (Additional file 1: Table S5), 1.0 μL of 25× SYBR green, 4 μL nuclease-free water, and 12 μL of DNA template for a 30 μL PCR reaction. PCR cycling consisted of an initial incubation at 72 °C for 3 min then 95 °C for 30 s and cycling at 95 °C for 10 s, 63 °C for 30 s, and 72 °C for 3 min until curves reach saturation. Generally reactions were stopped at the end of cycles 9 to 11. The products of the PCR reactions were visualized and quantitated with a UV-transilluminator after running 1 μL of PCR product on a 6 % Tris-borate-EDTA (TBE) gel for 20 min at 250 V and staining with SYBR gold. Barcoded samples were pooled and gel size selected for fragments between approximately 220-1,000 base pairs. Now libraries were ready for sequencing.

### Primary data processing

Sequencing was performed on HiSeq with 50 × 6 single end reads with single 6 base pair indexing for THS-seq data, and 50 × 8 × 8 single end reads with dual 8 base pair indexing for ATAC-seq data. Technical replicates were combined before analysis, while biological replicates were kept separate. Any down-sampling of mapped alignments was performed with in-house scripts randomly sampling reads from the original datasets. Sequencing reads were first mapped to the hg19 reference genome with BWA 0.7.5a-r405 using default parameters. After conversion to a bam file with samtools, clonal read removal was performed. For identifying significantly enriched regions, Dfilter was used with default parameters, unless stated. Overlaps were calculated using the bedtools function coverageBed. Individual loci read density data were extracted from BAM files, and converted to wig files and examined as a custom tract on the UCSC genome browser. Correlations were performed and graphed in R, and peak distribution graphs among other graphs were graphed with graphpad prism version 5.0. Proportional Venn diagrams were generated with Venn Diagram Plotter, (http://omics.pnl.gov/software/venn-diagram-plotter). Gene ontology analyses on significantly called peaks were performed with GREAT [28].

### Counting the number of alignments in peaks

Files with significantly called peaks generated by Dfilter were used to obtain peaks regions called for each sample. To count all alignments in peaks, the function ‘samtools view –Lh bam_files bed_region > > output’ was used to output and concatenate alignments overlapping the input BED peaks file to a new file, followed by counting the number of reads total in the outputted file. To count alignments in each individual peak, the function ‘samtools view –Lh bam_files bed_region > output’ was used for each individual peak, and then the number of alignments in each peak region was counted. To get the normalized number of reads per 100 base pairs, first any peaks that had zero alignments were removed, and then the total number of reads for each peak was divided by 100. Each dataset was sorted first by the number of reads per 100 base pairs, and then by peak length before plotting in Excel or GraphPad Prism version 5.0. For calculating the mean ± SEM, datasets with the normalized number of reads per 100 base pairs were saved as CSV files with peak length in the first column, and reads per 100 base pairs in the second column, and then run through an in-house Python script calculating the mean ± SEM for each peak length, with each peak length being in increments of 100 base pairs. Results were then graphed in GraphPad Prism version 5.0.

### Published ENCODE data analysis

Duke and UW ENCODE GM12878 lymphoblastoid datasets were downloaded from the UCSC genome browser (data accessible at NCBI GEO database [33], accessions GSM816665, GSM736496, GSM736620) and were processed using the same pipeline used to process THS-seq and ATAC-seq data, except bowtie version 1.0.0 with stringent parameters (bowtie -n1 -k1 --best --chunkmbs 10240 --strata -l32 -m1 -p4 --nomaqround –sam) was used for mapping. To get the most comprehensive accessibility data from Duke and UW, all replicates from each respective dataset were combined before mapping. In total, 144,738,228 and 47,417,059 total unique alignments were used for analysis for Duke and UW, respectively.

### Published ATAC-seq data analysis

All raw paired end ATAC-seq data for 50,000 cells and 500 cells was downloaded from GEO Gene Expression Omnibus (data accessible at NCBI GEO accession GSE47753). Only read 1 of the paired-end data was used for analysis to provide a fair comparison to single end THS-seq data. ATAC-seq read 1 datasets were then aligned with BWA using default parameters. Down-sampling of mapped alignments was performed with in-house scripts randomly sampling reads from the original datasets, and then run through Dfilter for calling of significant peaks.

### Ethics approval

No ethical approval was required for this study.

### Availability of data and materials

The data discussed in this publication have been deposited in NCBI’s Gene Expression Omnibus [33] (deposited 2015-08-14) and are accessible through GEO Series accession number GSE72089 (http://www.ncbi.nlm.nih.gov/geo/query/acc.cgi?acc=GSE72089).

## Additional file

Additional file 1: Figure S1. Schematic of transposon design and transposome complex generation. **Figure S2.** Validation of successful transposon insertion and DNA fragmentation. **Figure S3.** Validation of T7 *in vitro* transcription. **Figure S4.** Validation of PCR amplification for barcode addition. **Figure S5.** Tn5059 concentration and dimethylformamide titrations for optimal *in vitro* transcription amplification yields. **Figure S6.** Comparison of peak region distances to transcription start sites. **Figure S7.** Significant gene ontology biological processes categories for each peaks dataset. **Figure S8.** Quantitation of mitochondrial reads in all datasets. **Figure S9.** 100-cell THS-seq/Tn5059 read statistics analysis. **Figure S10.** Peak overlap comparisons in all datasets. **Figure S11.** Base pair overlap comparisons in all datasets. **Figure S12.** Examination of peak overlap in a pairwise comparison of all experimental datasets. **Figure S13.** Examination of base pair overlap in a pairwise comparison of all experimental datasets. **Figure S14.** Two hundred kilobyte view of accessible chromatin marks in 500-cell datasets. **Figure S15.** Overview of chromatin accessibility at gene loci enriched in 100- and 500-cell THS-seq/Tn5059 data and not enriched in 500-cell ATAC-seq/EzTn5 data. **Figure S16.** Overview of chromatin accessibility at major genes implicated in cancer and in immune system function. **Figure S17.** Peak size distribution comparisons between THS-seq, ATAC-seq, and ENCODE data. **Figure S18.** Validation of peaks based on peak length. **Figure S19.** THS-seq and ATAC-seq peak capture preferences. **Figure S20.** Comparison of 500-cell ATAC-seq/EzTn5 data to published ATAC-seq data. **Figure S21.** Two hundred kilobyte view of accessible chromatin in ATAC-seq/EzTn5 data versus published ATAC-seq data. **Table S1.** 100-cell THS-seq/Tn5059 data and controls with read statistics and data analysis. **Table S2.** Peak size counts versus lengths of Dfilter called peaks for each sample. **Table S3.** Analysis of datasets down-sampled to 8,351,125 unique reads and analysis of published ATAC-seq datasets. **Table S4.** Original datasets analysis results. **Table S5.** THS-seq oligo sequences.
