# Supplementary material for: Characterization of chromatin accessibility with a transposome hypersensitive sites sequencing (THS-seq) assay
Source: Genome Biol. 2016 Feb 4;17:20. doi: 10.1186/s13059-016-0882-7 (PMC4743176; doi:10.1186/s13059-016-0882-7)
Supplement: Additional file 1: Figure S1. — Schematic of transposon design and transposome complex generation. Figure S2. Validation of successful transposon insertion and DNA fragmentation. Figure S3. Validation of T7 in vitro transcription. Figure S4. Validation of PCR amplification for barcode addition. Figure S5. Tn5059 concentration and dimethylformamide titrations for optimal in vitro transcription amplification yields. Figure S6. Comparison of peak region distances to transcription start sites. Figure S7. Significant gene ontology biological processes categories for each peaks dataset. Figure S8. Quantitation of mitochondrial reads in all datasets. Figure S9. 100-cell THS-seq/Tn5059 read statistics analysis. Figure S10. Peak overlap comparisons in all datasets. Figure S11. Base pair overlap comparisons in all datasets. Figure S12. Examination of peak overlap in a pairwise comparison of all experimental datasets. Figure S13. Examination of base pair overlap in a pairwise comparison of all experimental datasets. Figure S14. Two hundred kilobyte view of accessible chromatin marks in 500-cell datasets. Figure S15. Overview of chromatin accessibility at gene loci enriched in 100- and 500-cell THS-seq/Tn5059 data and not enriched in 500-cell ATAC-seq/EzTn5 data. Figure S16. Overview of chromatin accessibility at major genes implicated in cancer and in immune system function. Figure S17. Peak size distribution comparisons between THS-seq, ATAC-seq, and ENCODE data. Figure S18. Validation of peaks based on peak length. Figure S19. THS-seq and ATAC-seq peak capture preferences. Figure S20. Comparison of 500-cell ATAC-seq/EzTn5 data to published ATAC-seq data. Figure S21. Two hundred kilobyte view of accessible chromatin in ATAC-seq/EzTn5 data versus published ATAC-seq data. Table S1. 100-cell THS-seq/Tn5059 data and controls with read statistics and data analysis. Table S2. Peak size counts versus lengths of Dfilter called peaks for each sample. Table S3. Analysis of datasets down-sampled to 8,351,125 unique re [file 13059_2016_882_MOESM1_ESM.pdf]

Supplementary File	Title
Supplementary Figure 1	Schematic of transposon design and transposome complex generation
Supplementary Figure 2	Validation of successful transposon insertion and DNA fragmentation
Supplementary Figure 3	Validation of T7 *in vitro* transcription
Supplementary Figure 4	Validation of PCR amplification for barcode addition
Supplementary Figure 5	Tn5059 concentration titrations and titration of dimethylformamide concentrations for optimal *in vitro* transcription amplification yields
Supplementary Figure 6	Comparison of peak region distances to transcription start starts.
Supplementary Figure 7	Significant gene ontology biological processes categories for each peaks dataset
Supplementary Figure 8	Quantitation of mitochondrial reads in all datasets
Supplementary Figure 9	100-cell THS-seq/Tn5059 read statistics analysis to determine minimal number of raw reads for quality data
Supplementary Figure 10	Peak overlap comparisons in all datasets
Supplementary Figure 11	Base pair overlap comparisons in all datasets
Supplementary Figure 12	Examination of peak overlap in a pairwise comparison of all experimental datasets
Supplementary Figure 13	Examination of base pair overlap in a pairwise comparison of all experimental datasets
Supplementary Figure 14	200 kb view of accessible chromatin marks in 500 cell datasets

Supplementary Figure 15	Overview of chromatin accessibility at gene loci enriched in 100- and 500-cell THS-seq data with Tn5059 and not enriched in 500-cell ATAC-seq/EzTn5 data
Supplementary Figure 16	Overview of chromatin accessibility at major genes implicated in cancer and in immune system function
Supplementary Figure 17	Peak size distribution comparisons between THS-seq, ATAC-seq and ENCODE data
Supplementary Figure 18	Validation of peaks based on peak length
Supplementary Figure 19	THS-seq and ATAC-seq peak capture preferences
Supplementary Figure 20	Comparison of 500-cell ATAC-seq/EzTn5 data to published ATAC-seq data
Supplementary Figure 21	200 kb view of accessible chromatin in ATAC-seq/EzTn5 data versus published ATAC-seq data
Supplementary Table 1	100-cell THS-seq/Tn5059 data and controls with read statistics and data analysis
Supplementary Table 2	Peak size counts versus lengths of Dfilter called peaks for each sample
Supplementary Table 3	Analysis of datasets down sampled to 8,351,125 unique reads and analysis of published ATAC-seq datasets
Supplementary Table 4	Original datasets analysis results
Supplementary Table 5	THS-seq oligo sequences

Supplementary Figure 1 description:

Schematic of transposon design and transposome complex generation. Transposon consists of the T7 promoter, Read primer sequence, and the mosaic end sequence for Tn5 binding that is contained within the read sequence.

Supplementary Figure 2 description:

Validation of successful transposon insertion and DNA fragmentation. The first two lanes are reactions using 0.7 µM Tn5059, the third and fourth lanes are reactions containing 0.5 µM EzTn5, the fifth and sixth lanes have Tn5 replaced with nuclease free water, the seventh lane is a reaction with 0.7 µM Tn5059 and with water substituted for DNA in the reaction, and the eighth lane is a reaction with 0.5 µM EzTn5 with water substituted for DNA in the reaction. The customized EzTn5 and Tn5059 transposomes are designed to fragment DNA upon transposon incorporation. By fragmenting DNA, this implies the mosaic end DNA oligo and transposon oligo anneal correctly, the transposome complex is forming correctly, the transposome complex is active, and the transposome complex can successfully fragment DNA and insert the transposon. Successful attachment of the transposon is indicated by generation of *in vitro* transcribed RNA, which is shown in Supplementary Figure 3. It is not expected to have dark smears for fragmented DNA, since 6 nanograms of DNA were used as input and no PCR was done after fragmentation. Experimentally a transposition reaction was performed on samples, cleaned with zymo DNA clean and concentrator, and then run on a 6% Tris-borate-EDTA (TBE) gel.

Supplementary Figure 3 description:

Validation of T7 *in vitro* transcription. 6% Tris-Borate-Urea (TBU) gels assaying for single stranded nucleic acids, in this case RNA, are used for validation of RNA amplification from *in vitro* transcription. Generally smears are seen from ~150 base pairs to >2000 base pairs. (a) RNA smears from THS-seq/Tn5059 using 100 cells, 1000 cells and 6 nanograms of pure DNA. No RNA smear is generated for the NTC control. (b) Quantitation of the *in vitro* transcribed RNA smears present in (a). (c) RNA smears are also present for assaying with both 500-cell THS-seq/Tn5059 and 500-cell THS-seq with Ez-Tn5 and 6 nanograms of pure DNA with each respective enzyme. No RNA smears are generated for the NTC controls for each respective enzyme. (d) Quantitation of the *in vitro* transcribed RNA smears present in (c).

Supplementary Figure 4 description:

Validation of PCR amplification for barcode addition. 6% TBE gels for assaying double stranded DNA are used here for confirming generation of barcoded libraries. (a) Libraries have been successfully generated for THS-seq/Tn5059 using 100 cells, 1000 cells and with 6 nanograms of pure DNA. No DNA smears are generated for the NTC control. (b) Quantitation of size selected library smears present in (a). (c) DNA smears are also present for assaying with both 500-cell THS-seq/Tn5059 and 500-cell THS-seq with Ez-Tn5 and 6 nanograms of pure DNA with each respective enzyme. No DNA smears are generated for the NTC controls for each respective enzyme. (d) Quantitation of size selected library smears present in (c). Subsequently libraries are pooled, and gel size selected between ~220-250 to ~1000 base pairs to remove primers and primer dimers below the 220-250 base pair range.

Supplementary Figure 5 description:

Tn5059 concentration titrations and titration of dimethylformamide concentrations for optimal *in vitro* transcription amplification yields. (a-d) Concentration titrations were performed to determine the optimal concentration of Tn5059 for assaying accessible chromatin on 1000 GM12878 lymphoblastoid cells that gives the most *in vitro* transcription amplification product. 6% TBU gels were used for visualization. (a,b) Concentration titration of Tn5059 was performed at a range of 3.5 μM to 0.2 μM Tn5059 final concentration in reaction. Here 0.9 μM Tn5059 gave the best amplification and the reaction with 3.5 μM Tn5059 was similar, however the smear looks washed out and not how IVT smears usually appear, also with 0.9 μM Tn5059 reagents are saved. The 1.8 μM Tn5059 reaction possibly had pipetting errors. (c,d) The concentration titration of Tn5059 was performed with 0.7 μM to 0.1 μM Tn5059 final in reaction, and here 0.7 μM gave the strongest amplification. The quantitation of IVT RNA differs greatly between the gels since differences in how the ladders ran affected quantitation, however the trends are similar. We also performed a titration on DMF to determine the optimal concentration for tagmentation that gives the most *in vitro* transcription amplification product. Reactions used 1000 GM12878 lymphoblastoid cells. Titration was performed on a transposon that was 17 base pairs longer than the one used in the study, T7tspn-top2, however the result of this titration should be applicable to any transposon that is being used to assay accessible chromatin. The concentration of other tagmentation reaction buffer components that was kept consistent while DMF concentrations were varied was, 165 mM Tris-OAc, pH 7.8, 330 mM K-OAc, 50 mM Mg-OAc. All reactions were carried out in 5 μL solutions. (e) First a wider range of DMF concentrations were titrated, and once an optimal concentration was determined, (f) a narrower range was titrated to confirm the results.

Supplementary Figure 6 description:

Comparison of peak region distances to transcription start sites. For peak calling all samples were down sampled to 8,351,125 unique reads. Peak files generated by Dfilter were inputted into GREAT for analysis. Peak regions that caused database errors were removed, which was usually one to ten peaks per file, and thus should not impact the analysis. The replicate represented in each analysis was chosen based on if they had more base pairs called significant, and more base pair overlap with ENCODE data. Datasets represented are: (a) 100-cell THS-seq/Tn5059 Rep2, (b) 500-cell THS-seq/Tn5059 Rep1, (c) 500-cell THS-seq/EzTn5 Rep1, (d) 500-cell ATAC-seq/Tn5059 Rep2, (e) 500-cell ATAC-seq/EzTn5 Rep2.

Supplementary Figure 7 description:

Significant gene ontology biological processes categories for each peaks dataset. This is part of the same analysis performed by GREAT that was used in Supplementary Figure 6, and thus the same peaks files are used as input. The replicate represented was chosen based on if they had more base pairs called significant, and more base pair overlap with ENCODE data. Datasets represented are: (a) 100-cell THS-seq/Tn5059 Rep2, (b) 500-cell THS-seq/Tn5059 Rep1, (c) 500-cell THS-seq/EzTn5 Rep1, (d) 500-cell ATAC-seq/Tn5059 Rep2, (e) 500-cell ATAC-seq/EzTn5 Rep2.

Supplementary Figure 8 description:

Quantitation of mitochondrial reads in all datasets. The number of mitochondrial reads (chrM) were counted and represented as a percentage of (a) the total number of clonal reads, and (b) the total number of mapped reads in each dataset. (c) In 100-cell THS-seq/Tn5059, the proportion of clonal reads from each chromosome was calculated as a percentage of the total clonal reads for each sample.

Supplementary Figure 9 description:

100-cell THS-seq/Tn5059 read statistics analysis to determine minimal number of raw reads for quality data. Raw reads were down sampled in increments of 10 million reads with in house scripts, run through BWA with default parameters, and then were run through Dfilter to (a) call peaks, (b) determine total number of base pairs called significant, (c) calculate ENCODE data overlap, and (d) determine the total number of unique and clonal reads. The mean and standard deviation between the two 100-cell THS-seq/Tn5059 replicates was calculated and displayed.

Supplementary Figure 10 description:

Peak overlap comparisons in all datasets. Peaks were counted as overlapped if at least one base pair is overlapped in two significant peaks that are overlapped against each other in the datasets being compared. All samples except ENCODE Duke and UW samples were down sampled to the same number of unique reads, 8,351,125, before peak calling. (a) Peak overlap for all experimental replicates when overlapped against each other. (b) Peak overlap for the experimental replicate with the most base pairs called significant against ENCODE Duke data. (c) Peak overlap for the experimental replicate with the most base pairs called significant against ENCODE UW data. (d) Peak overlap for the experimental replicate with the most base pairs called significant against published 50,000-cell ATAC-seq replicate 4 data. (e) Peak overlap of ENCODE UW and Duke data against themselves, and ENCODE UW and Duke data against published 50,000-cell ATAC-seq replicate 4 data.

Supplementary Figure 11 description:

Base pair overlap comparisons in all datasets. If a base pair that is within a significant peak is overlapped with the same base pair in a significant peak in the dataset being compared to, then it is called significant and counted. All samples except ENCODE Duke samples were down sampled to the same number of unique reads, 8,351,125, before peak calling. (a) Base pair overlap for all experimental replicates when overlapped against each other. (b) Base pair overlap for the experimental replicate with the most base pairs called significant against ENCODE Duke data. (c) Base pair overlap for the experimental replicate with the most base pairs called significant against published 50,000-cell ATAC-seq replicate 4 data. (d) Base pair overlap of the two most comprehensive published 50,000-cell ATAC-seq datasets, replicate 3 and replicate 4.

Supplementary Figure 12 description:

Examination of peak overlap in a pairwise comparison of all experimental datasets. Peaks were counted as overlapped if at least one base pair is overlapped in two significant peaks that are overlapped against each other, in the datasets being compared. All samples were down sampled to the same number of unique reads, 8,351,125, before peak calling.

Supplementary Figure 13 description:

Examination of base pair overlap in a pairwise comparison of all experimental datasets. If a base pair that is within a significant peak is overlapped with the same base pair in a significant peak in the dataset being compared to, then it is called significant and counted. All samples were down sampled to the same number of unique reads, 8,351,125, before peak calling.

Supplementary Figure 14 description:

200 kb view of accessible chromatin marks in 500 cell datasets. Comparison between THS-seq/Tn5059, THS-seq/EzTn5, ATAC-seq/Tn5059, and ATAC-seq/EzTn5 against ENCODE GM12878 lymphoblastoid cell chromatin accessibility data from Duke and UW, and against histone modifications which are often found near regulatory elements and promoters. All samples were down sampled to the same number of unique reads, 8,351,125, before viewing tracts.

Supplementary Figure 15 description:

Overview of chromatin accessibility at gene loci enriched in 100- and 500-cell THS-seq/Tn5059 data and not enriched in 500-cell ATAC-seq/EzTn5 data. Enriched genes from GREAT were used to determine which genes were enriched in THS-seq and not ATAC-seq. 100- and 500-cell THS-seq/Tn5059 and 500-cell ATAC-seq/EzTn5 were down sampled to the same number of unique reads, 8,351,125, before analysis. UW DNase chromatin accessibility data was used for comparison since it was the most comprehensive of the GM12878 ENCODE datasets. ENCODE histone modifications which are often found near regulatory elements and promoters are also depicted. The replicate with the highest number of base pairs called significant was used in the read density data tract for each sample and for analysis. Also under each tract, the regions called significant by Dfilter are represented by bars. The gene loci for (a) ABCA12, (b) FRCL4, (c) IFI44, (d) PDGFD, and (e) PHLDB2 are represented.

Supplementary Figure 16 description:

Overview of chromatin accessibility at major genes implicated in cancer and in immune system function. 100- and 500-cell THS-seq/Tn5059 data and 500-cell ATAC-seq/EzTn5 data were down sampled to the same number of unique reads, 8,351,125, before analysis. UW DNase chromatin accessibility data was used for comparison since it was the most comprehensive of the GM12878 ENCODE datasets. ENCODE histone modifications which are often found near regulatory elements and promoters are also depicted. The replicate with the highest number of base pairs called significant was used in the read density data tract for each sample and for

analysis. Also under each tract, the regions called significant by Dfilter are represented by bars. The gene loci for (a) BCL2, (b) CASP3, (c) CD38, (d) the interferon gene cluster and specifically IFNA2, and (e) MAPK1 are represented. Enrichments performed by GREAT reported BCL2, CD38 and MAPK1 in the B cell receptor signaling pathway gene ontology biological process category. BCL2, CD38 and MAPK1 were reported for the B cell homeostasis gene ontology biological process category. IFNA2, IFNA1, IFNA5, IFNA6 and IFNA8 were reported for the response to type I interferon gene ontology biological process category.

Supplementary Figure 17 description:

Peak size distribution comparisons between THS-seq, ATAC-seq and ENCODE data. ENCODE data from Duke and UW for chromatin accessibility on GM12878 lymphoblastoid cells was processed as described in methods. All THS-seq and ATAC-seq datasets were down sampled to the same number of unique reads, 8,351,125, before analysis, except for 100-cell THS-seq/Tn5059 datasets, and the published 500-cell ATAC-seq replicate two dataset, which had less than 8,351,125 unique reads. For all datasets, counts of the number of peaks for each peak size were performed in 100 base pair increments, using the peaks file generated by Dfilter. Some data points were excluded from the graphs because values were beyond the axis, and the number of data points excluded for each graph is: (a) 14, (b) 8, (c) 8, (d) 8, (e) 8, (f) none, (g) 8, (h) 11. Samples compared to ENCODE data include: (a) 100-cell THS-seq/Tn5059, (b) 500-cell THS-seq/Tn5059, (c) 500-cell THS-seq/EzTn5, (d) 500-cell ATAC-seq/Tn5059, (e) 500-cell ATAC-seq/EzTn5, (g) Published 50,000-cell ATAC-seq data, and (h) published 500-cell ATAC-seq data. (f) A peaks distribution comparison of 500-cell THS-seq/Tn5059 was also done against published 50,000-cell ATAC-seq data.

Supplementary Figure 18 description:

Validation of peaks based on peak length. (a) The percentage more peaks found in 100-cell and 500-cell THS-seq/Tn5059 data then in 500-cell ATAC-seq/EzTn5 data, and the percentage more 100-cell and 500-cell THS-seq/Tn5059 and UW base pair overlap then in 500-cell ATAC-seq/EzTn5. (b) Zoom in on graph (a) showing the peak lengths between 100-1200 base pairs. (c) The percentage more peaks found in 100-cell and 500-cell THS-seq/Tn5059 data then in 500-cell ATAC-seq/EzTn5 data, and the percentage more 100-cell and 500-cell THS-seq/Tn5059 and Duke base pair overlap then in 500-cell ATAC-seq/EzTn5. (d) Zoom in on graph (c) showing the peak lengths between 100-1200 base pairs. (e) The percentage more peaks found in 100-cell and 500-cell THS-seq/Tn5059 data then in 500-cell ATAC-seq/EzTn5

data, and the percentage more normalized 100-cell and 500-cell THS-seq/Tn5059 and Duke base pair overlap then in 500-cell ATAC-seq/EzTn5. Normalizing was performed using the global base pair overlap values for the ENCODE Duke dataset. (f) Zoom in on graph (e) showing the peak lengths between 100-1200 base pairs.

Supplementary Figure 19 description:

THS-seq and ATAC-seq peak capture preferences. All datasets were down sampled to 8,351,125 unique reads before analysis. Normalization was performed by calculating the number of reads per 100 base pairs in each peak, and any peaks with zero alignments were removed before analysis. (a-c) For all peaks in each individual dataset, the normalized number of alignments in each peak represented by mean ± SEM in (a) 500-cell THS-seq/Tn5059 and 500-cell ATAC-seq/EzTn5 (b) 100-cell and 500-cell THS-seq/Tn5059 (c) 100-cell THS-seq/Tn5059 and published 500-cell ATAC-seq data. Some data points were excluded from the graphs because values were beyond the axis, and the number of data points excluded for each graph is: (a) 13, (b) 4, and (c) 15. (d-h) For all peaks in each individual dataset, the normalized number of unique alignments in each peak, visualized from highest peak length to lowest, and with the most to the least number of normalized unique alignments for each peak length from left to right. Datasets represented had the most base pairs called significant by Dfilter of the two replicates for each condition. Datasets include (d) 100-cell THS-seq/Tn5059 Rep2, (e) 500-cell THS-seq/Tn5059 Rep1, (f) 500-cell THS-seq/EzTn5 Rep1, (g) 500-cell ATAC-seq/Tn5059 Rep2, and (h) 500-cell ATAC-seq/EzTn5 Rep2.

Supplementary Figure 20 description:

Comparison of 500-cell ATAC-seq/EzTn5 data to published ATAC-seq data. A modified protocol that included an optimized tagmentation buffer and protease digestion of proteins and transposase were used for generating 500-cell ATAC-seq/EzTn5 data, which was then compared to published 50,000-cell ATAC-seq data, and published 500-cell ATAC-seq data. All samples were down sampled to the same number of unique reads, 8,351,125, before analysis. The replicate with the highest number of base pairs called significant was used in the venn diagram representations for each sample and in analysis. Comparisons were made examining (a) the total number of peaks called, (b) total number of base pairs called significant, (c) base pair overlap of published 50,000-cell ATAC-seq data, 500-cell ATAC-seq/EzTn5 data and published 500-cell ATAC-seq data against ENCODE accessibility data from UW and (d) base

pair overlap of published 50,000-cell ATAC-seq data, 500-cell ATAC-seq/EzTn5 data and published 500-cell ATAC-seq data against ENCODE accessibility data from Duke.

Supplementary Figure 21 description:

200 kb view of accessible chromatin in ATAC-seq/EzTn5 data versus published ATAC-seq data. 500-cell ATAC-seq/EzTn5 data was compared to published 50,000-cell ATAC-seq data, and published 500-cell ATAC-seq data. 500-cell ATAC-seq/EzTn5 data was generated using modified protocols with optimized tagmentation buffer and protease digestion of proteins and transposase. ENCODE accessibility data and histone modifications which are often found near regulatory elements and promoters are also depicted. All samples were down sampled to the same number of unique reads, 8,351,125, before viewing tracts.

Supp. Figure 1

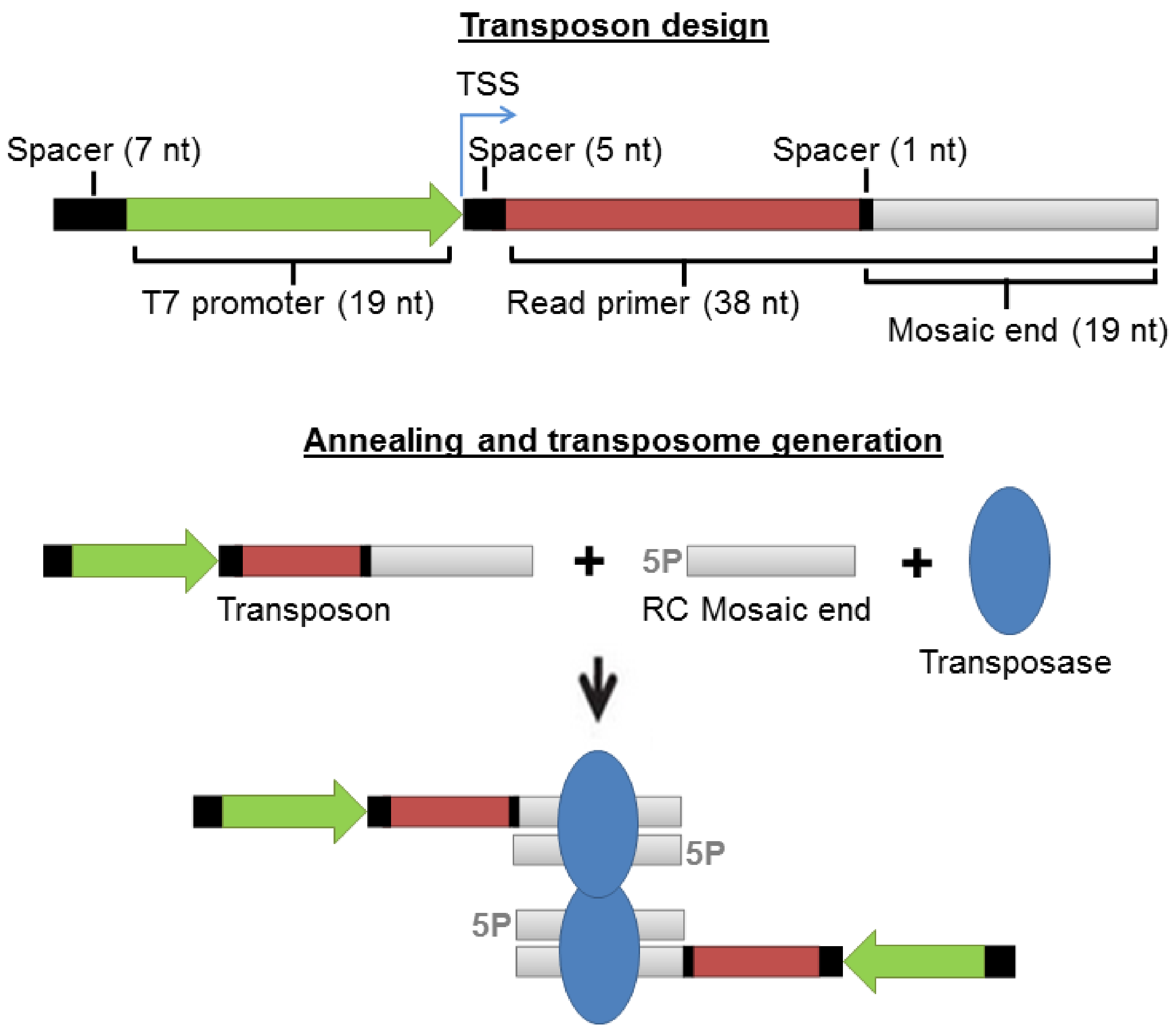

Supp. Figure 2

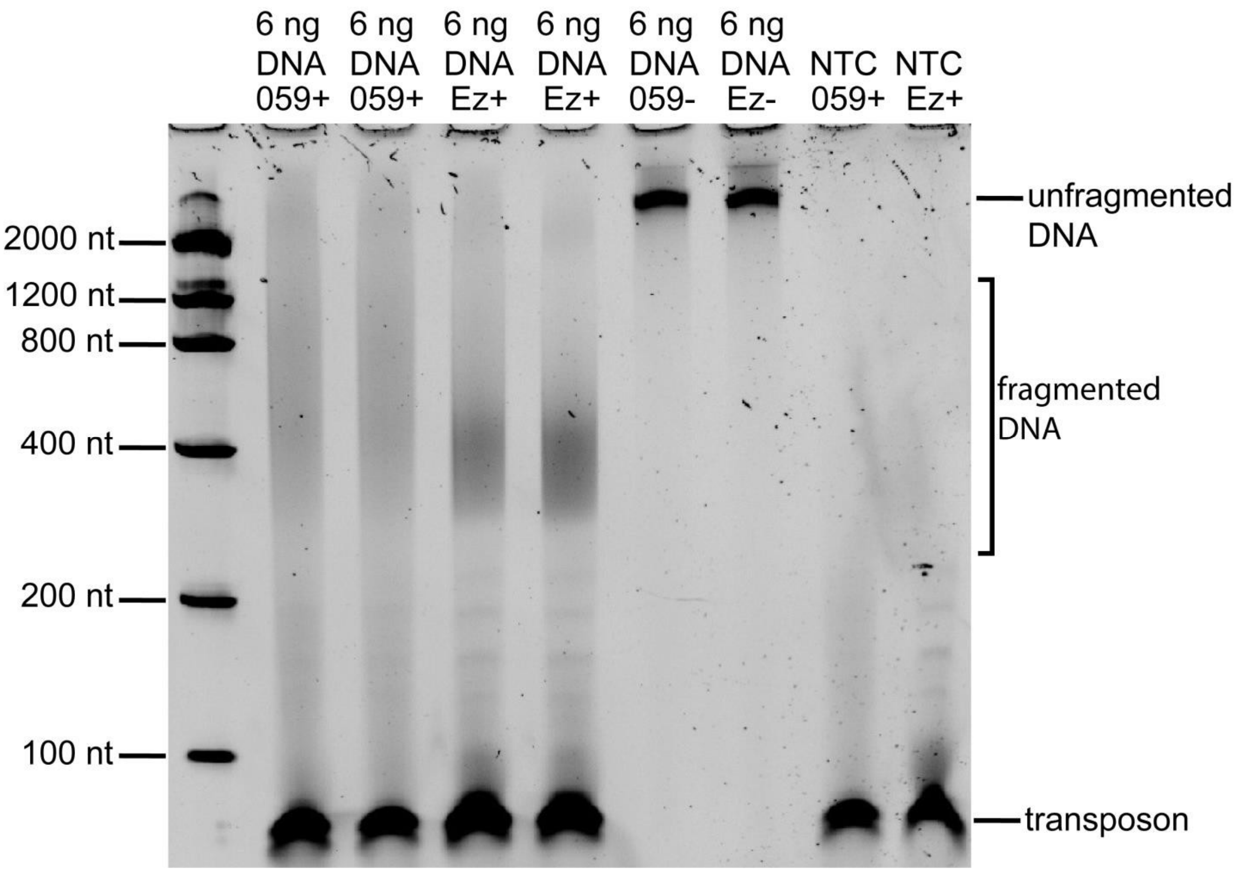

Supp. Figure 3

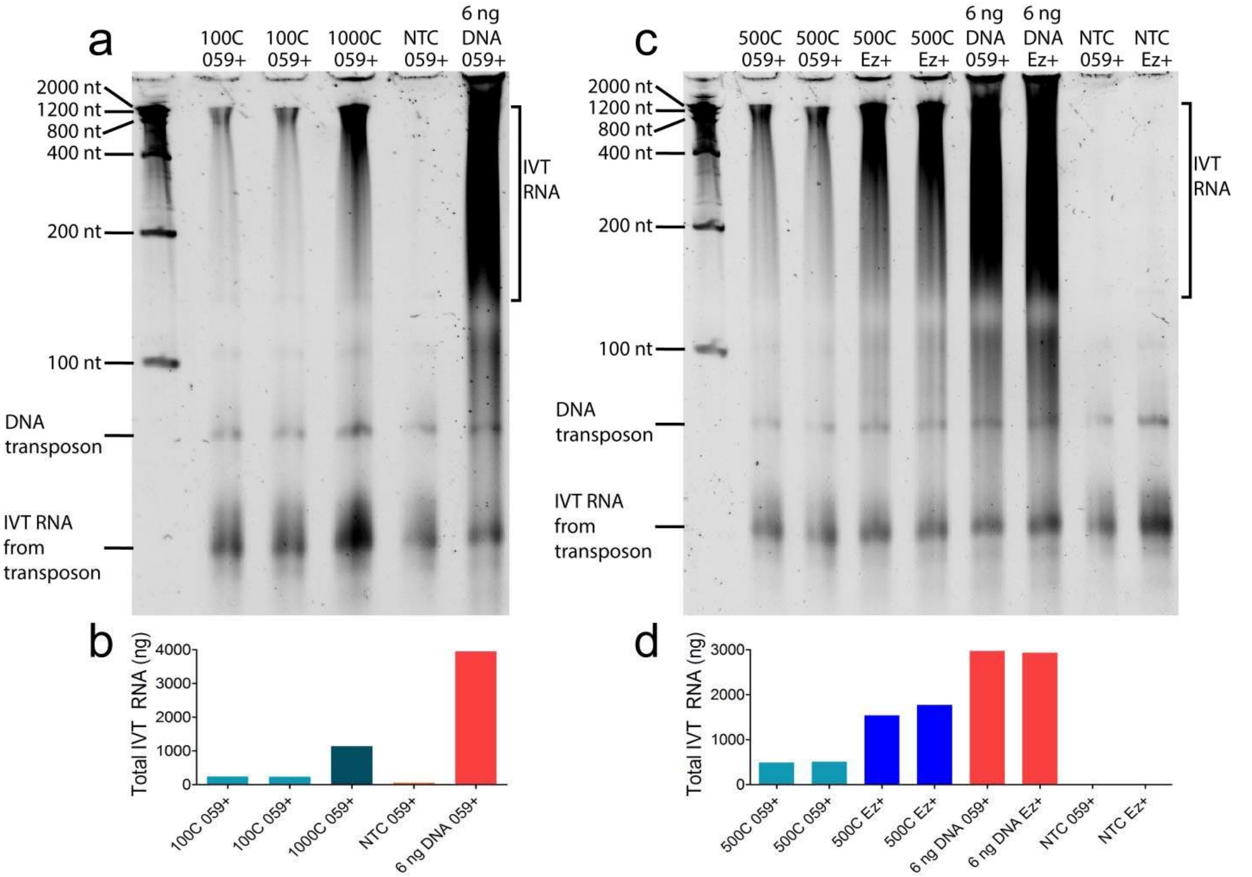

Supp. Figure 4

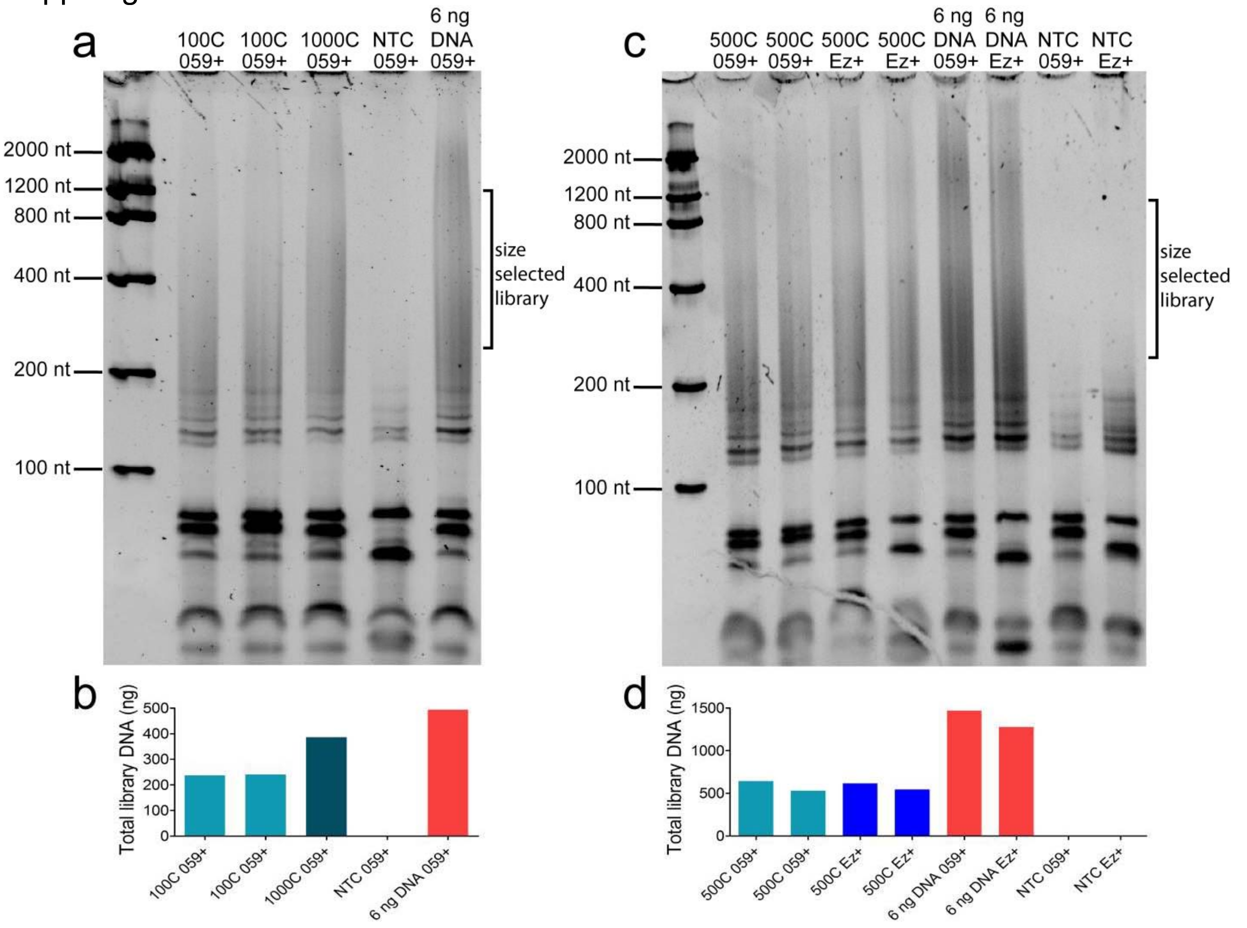

Supp. Figure 5

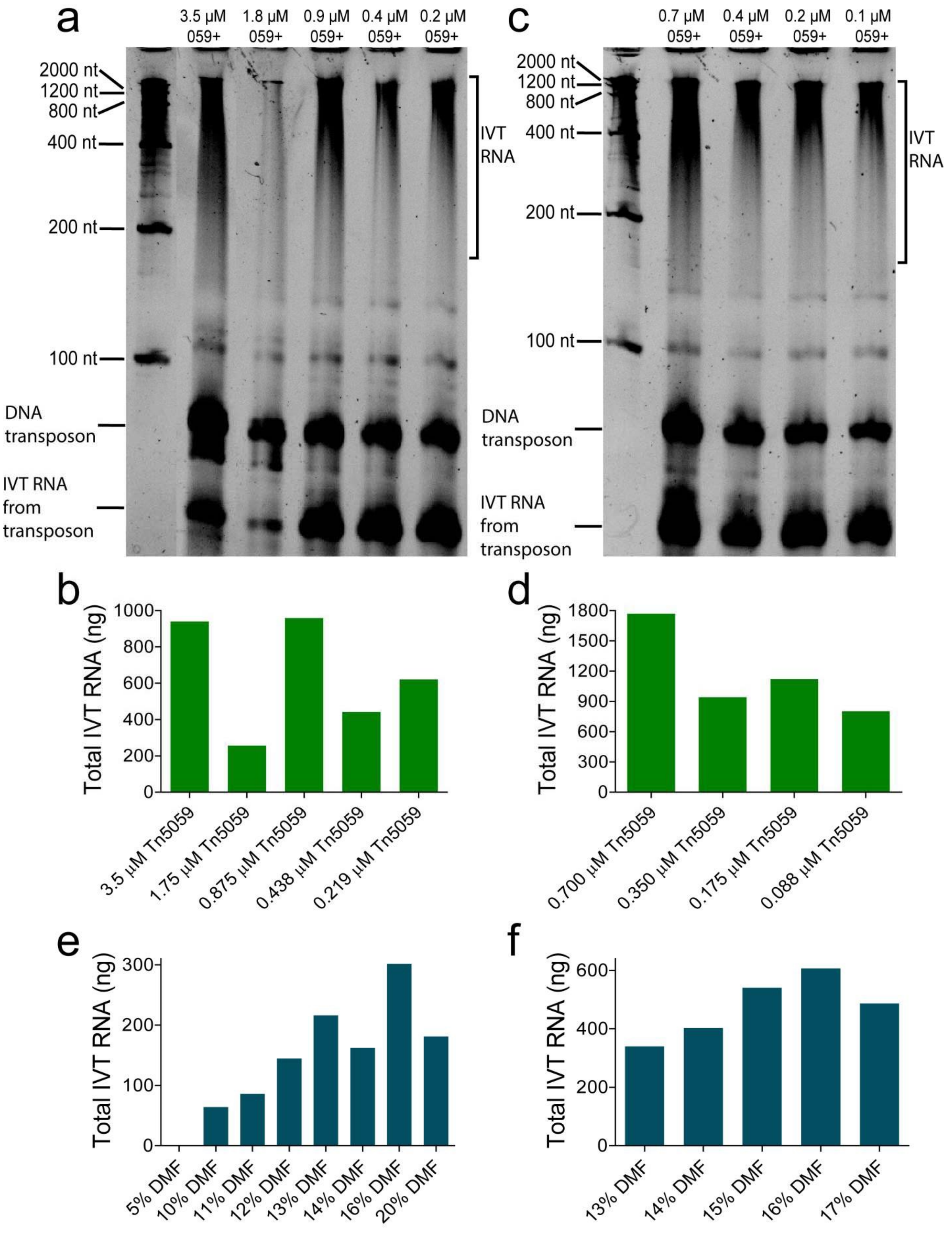

Supp. Figure 6

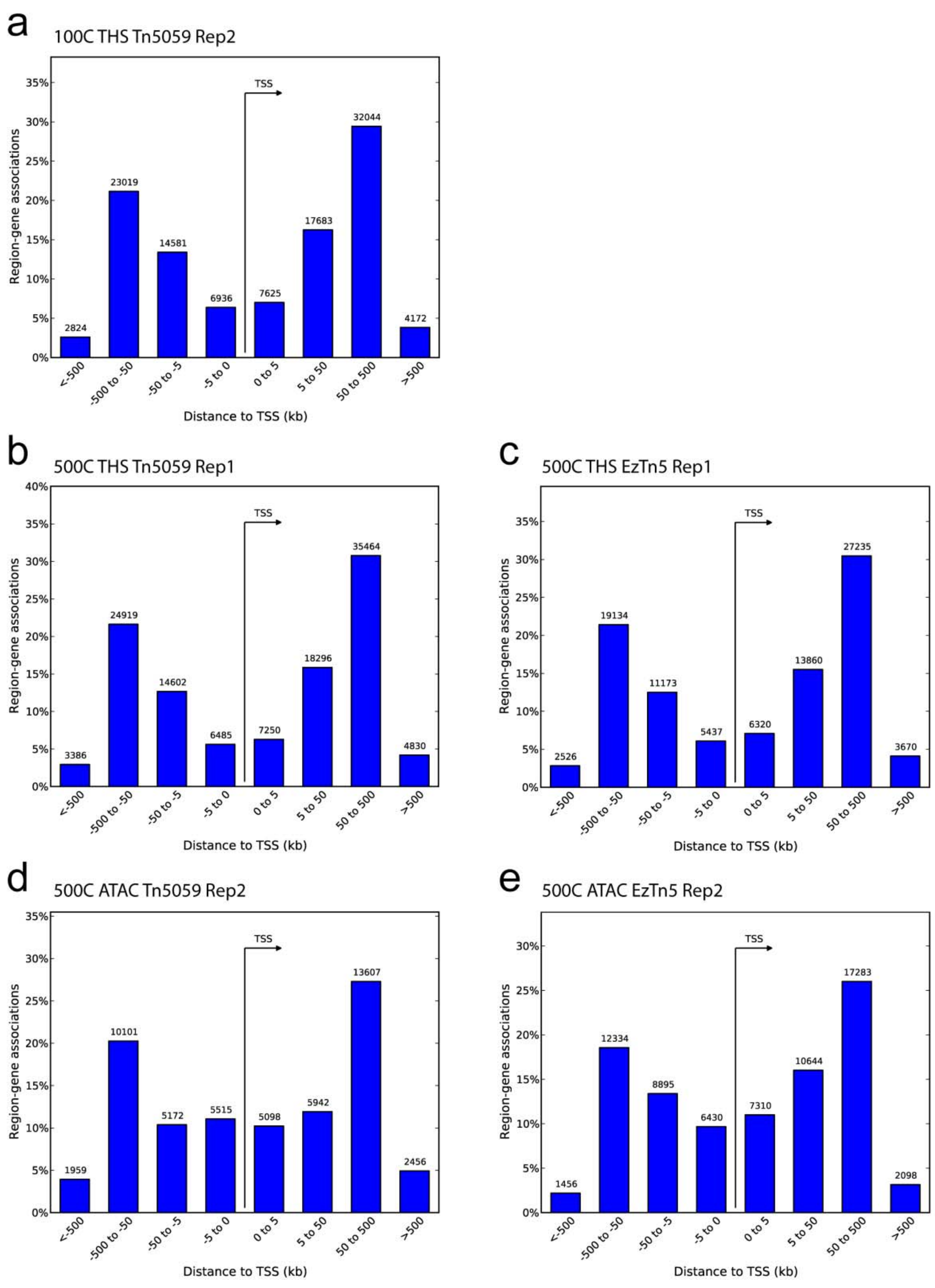

Supp. Figure 7

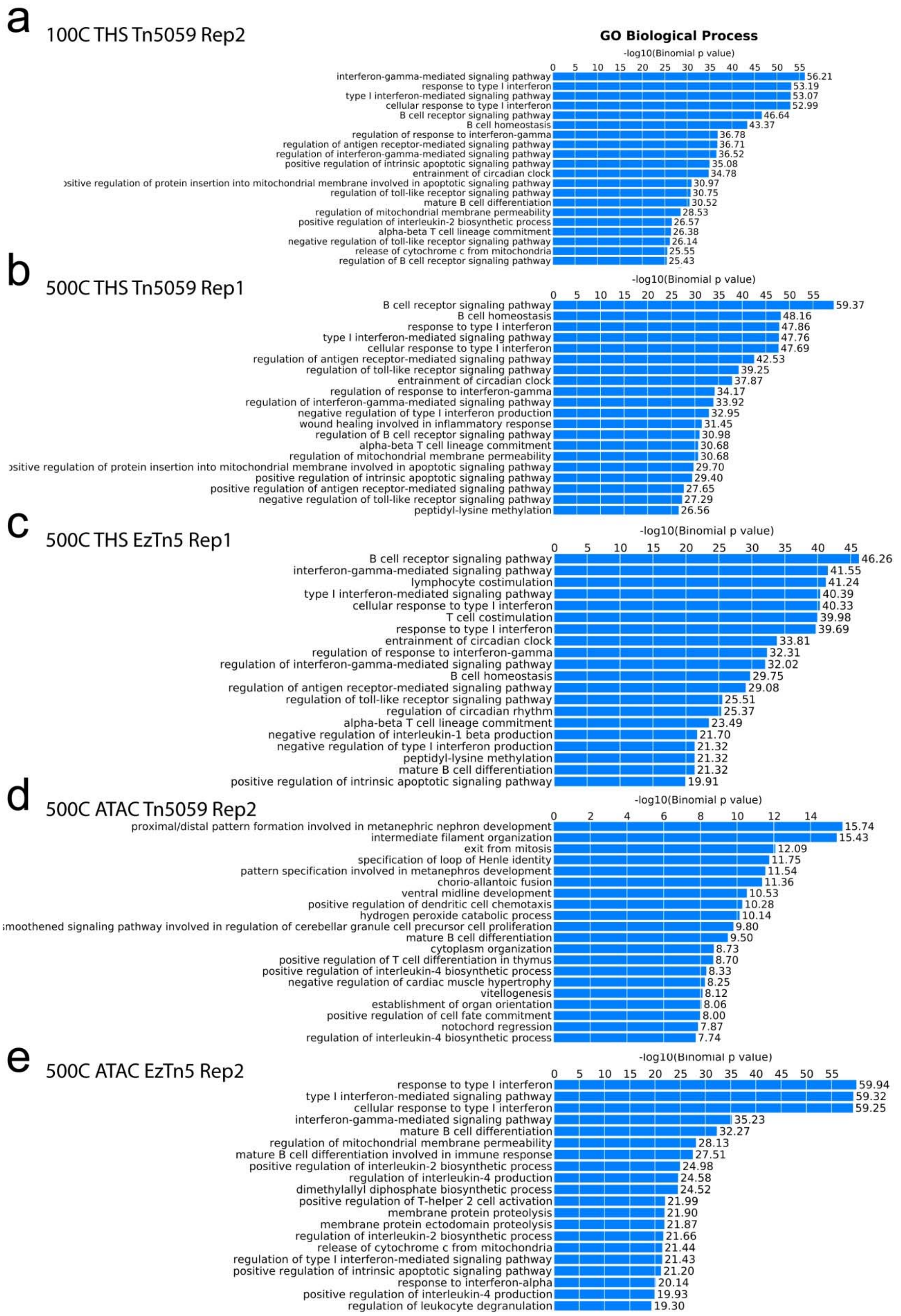

Supp. Figure 8

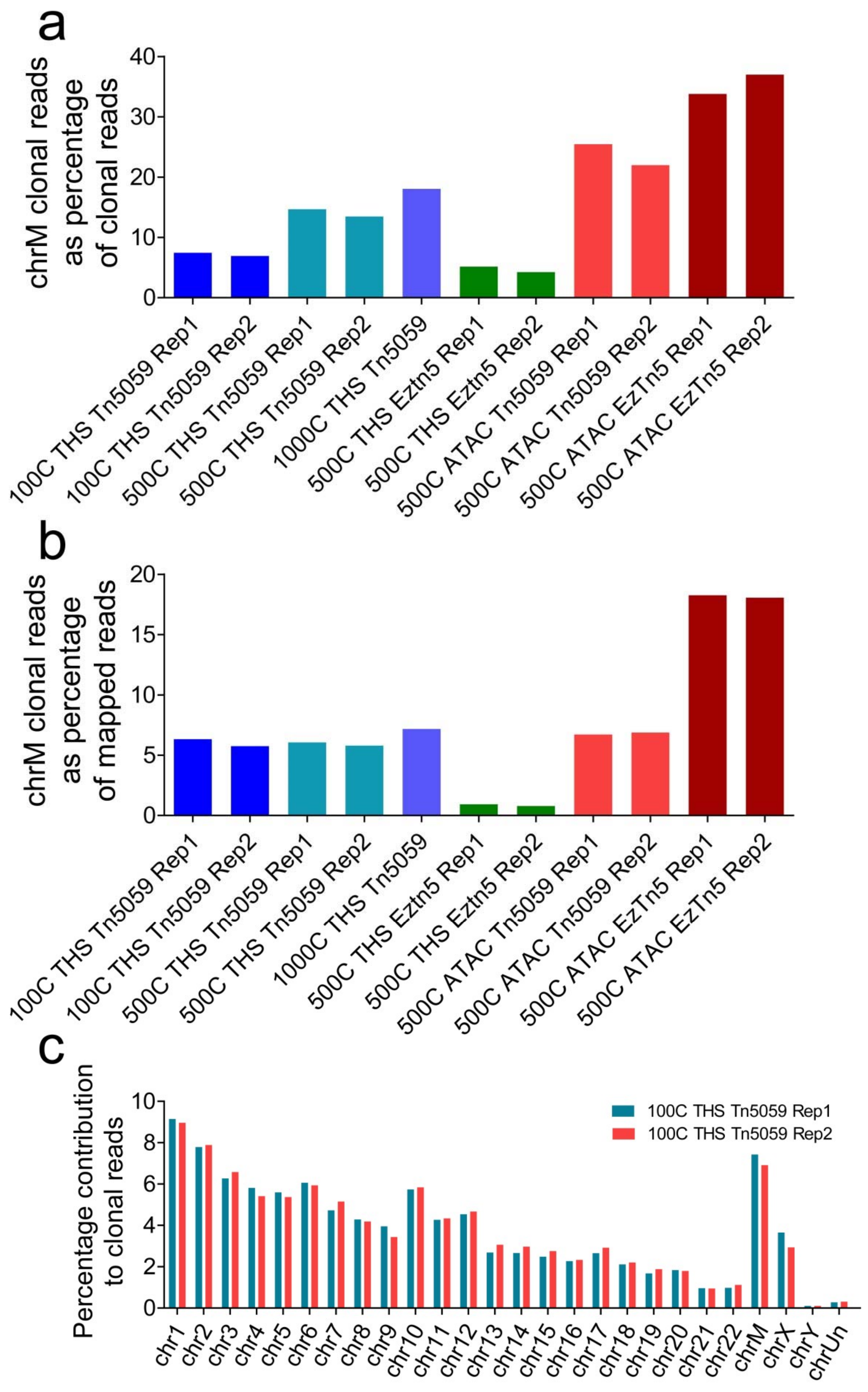

Supp. Figure 9

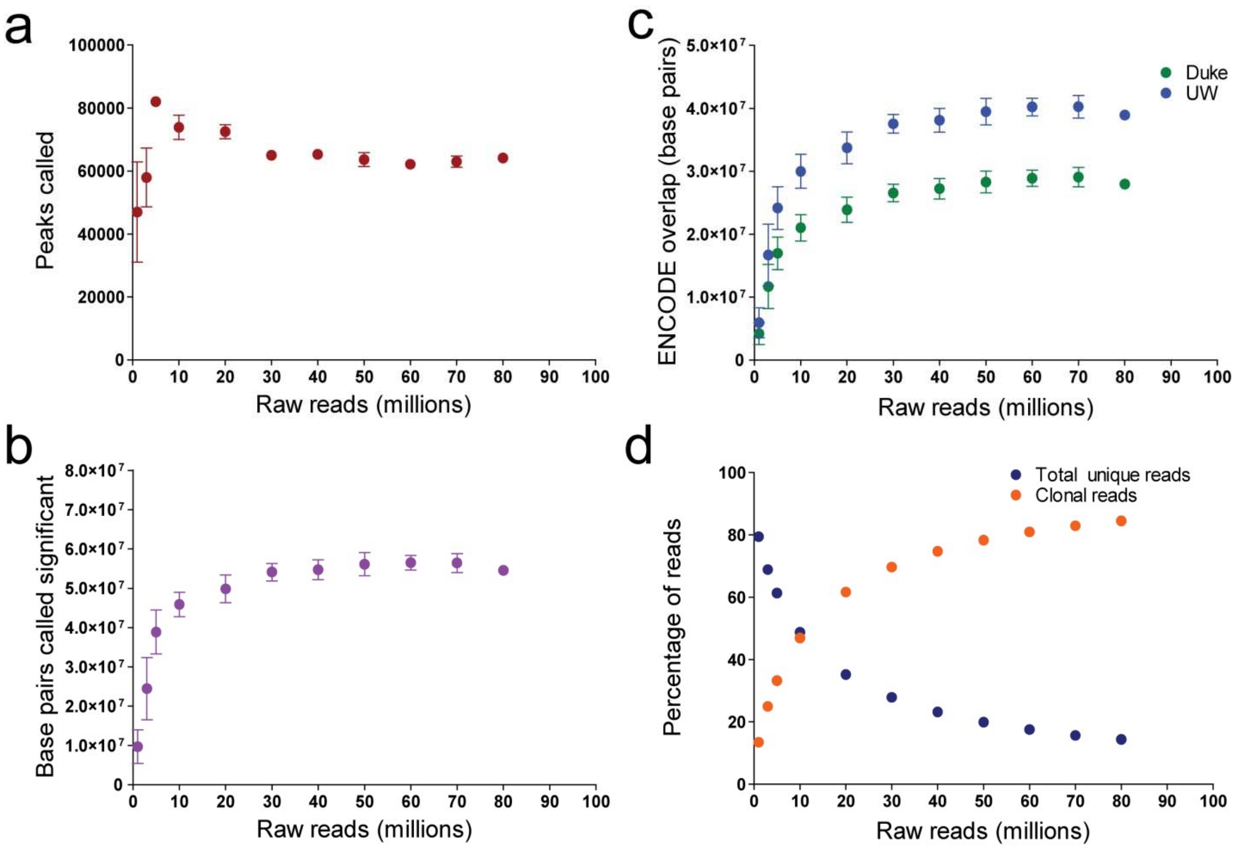

Supp. Figure 10

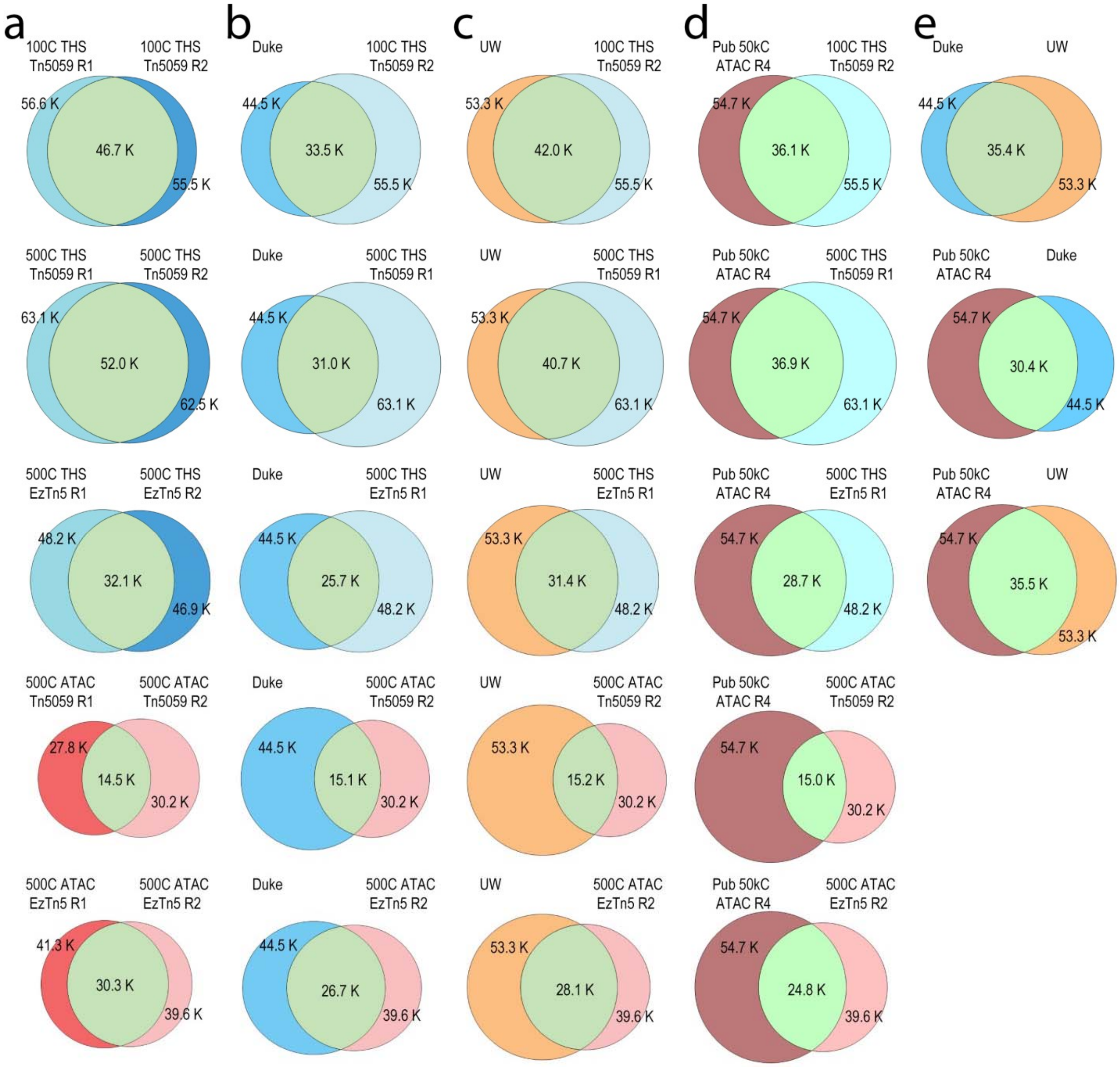

Supp. Figure 11

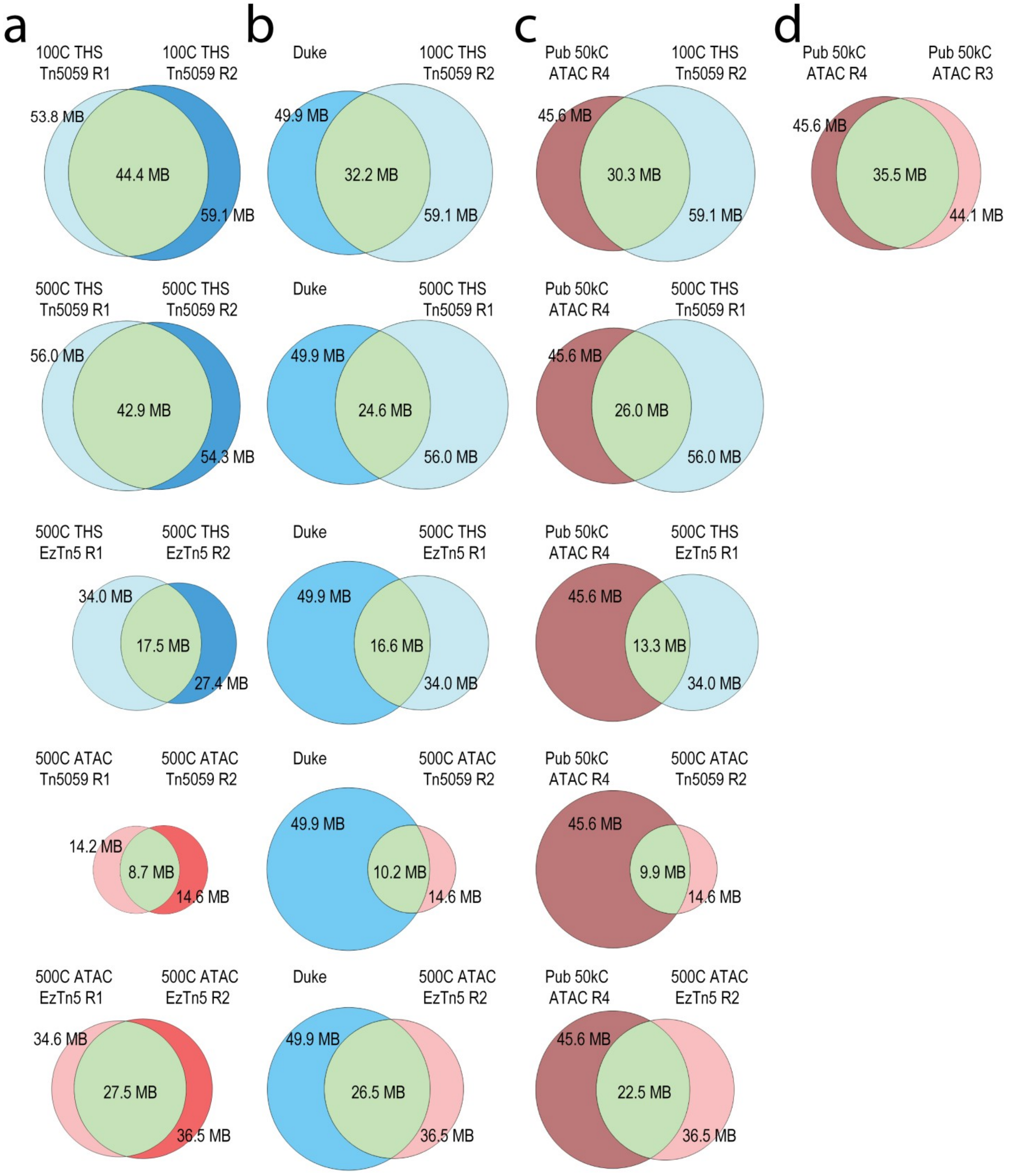

Supp. Figure 12

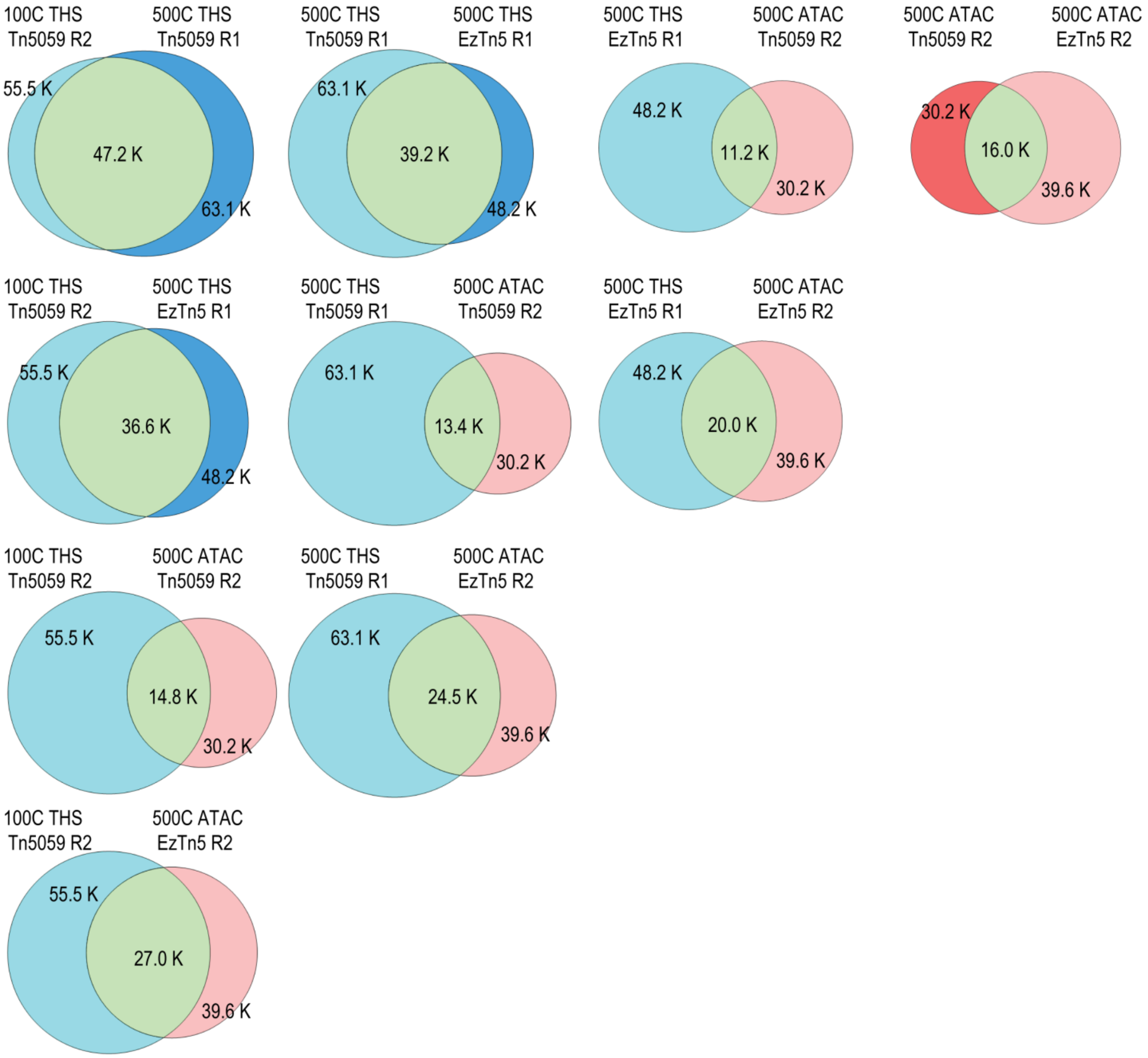

Supp. Figure 13

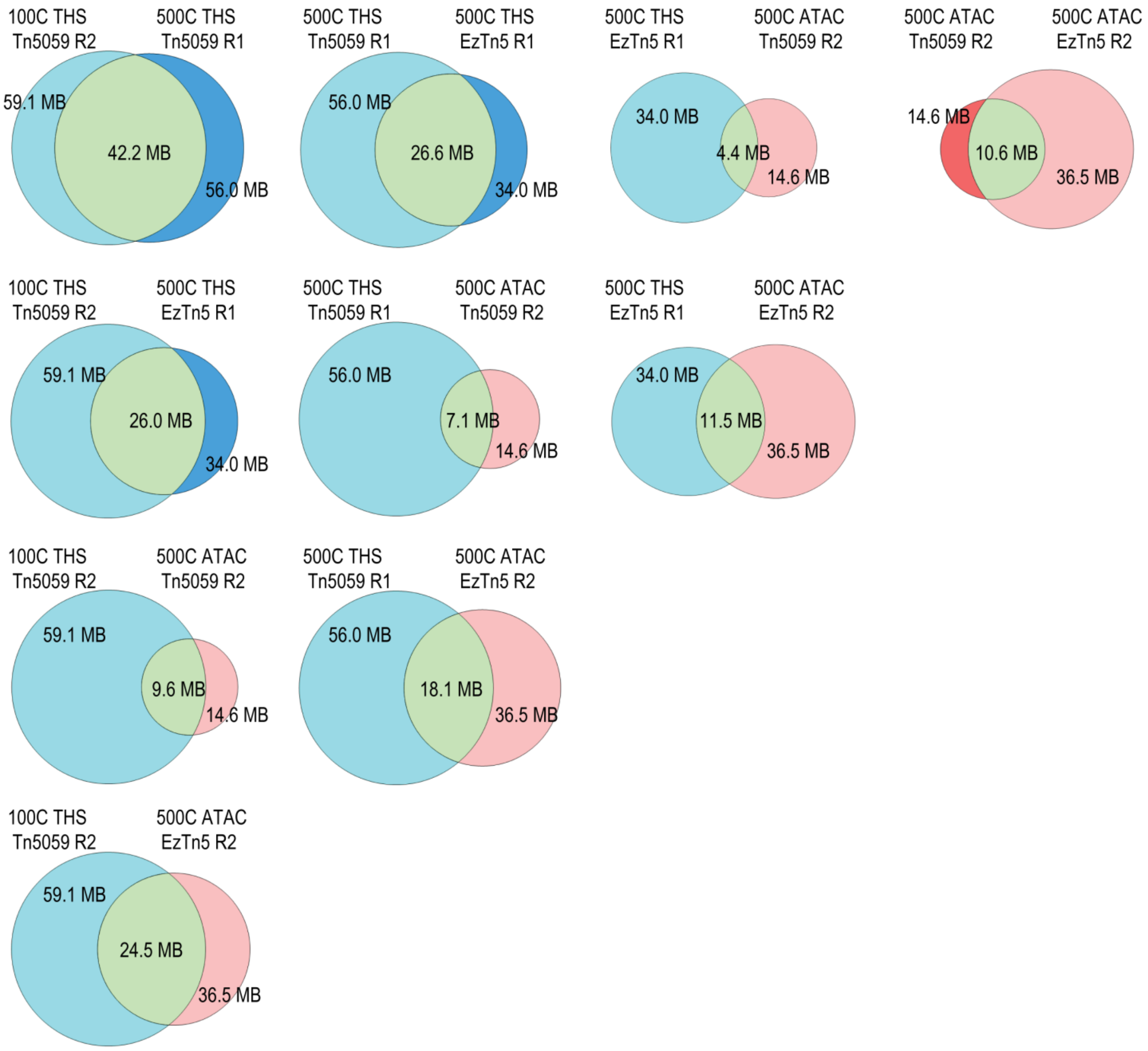

Supp. Figure 14

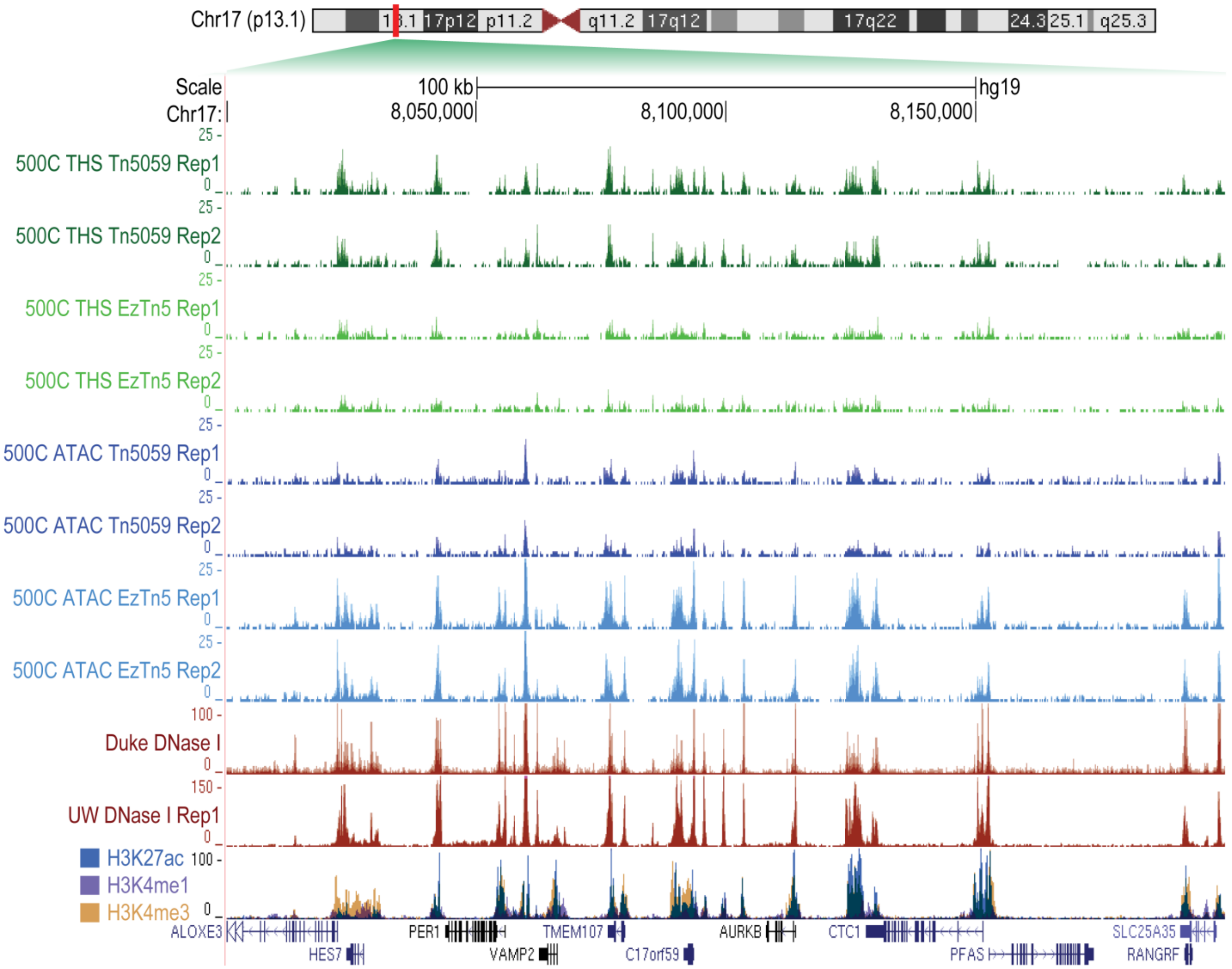

Supp. Figure 15

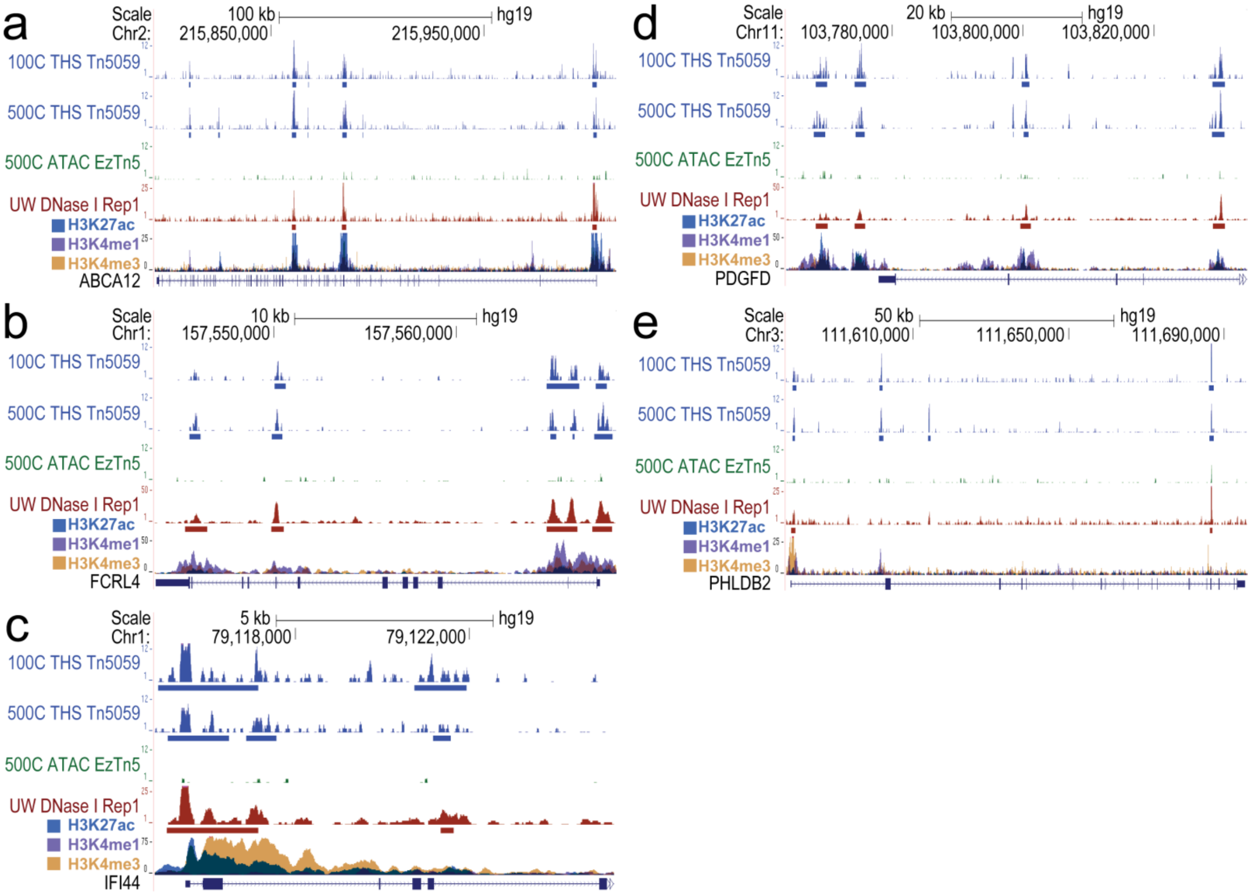

Supp. Figure 16

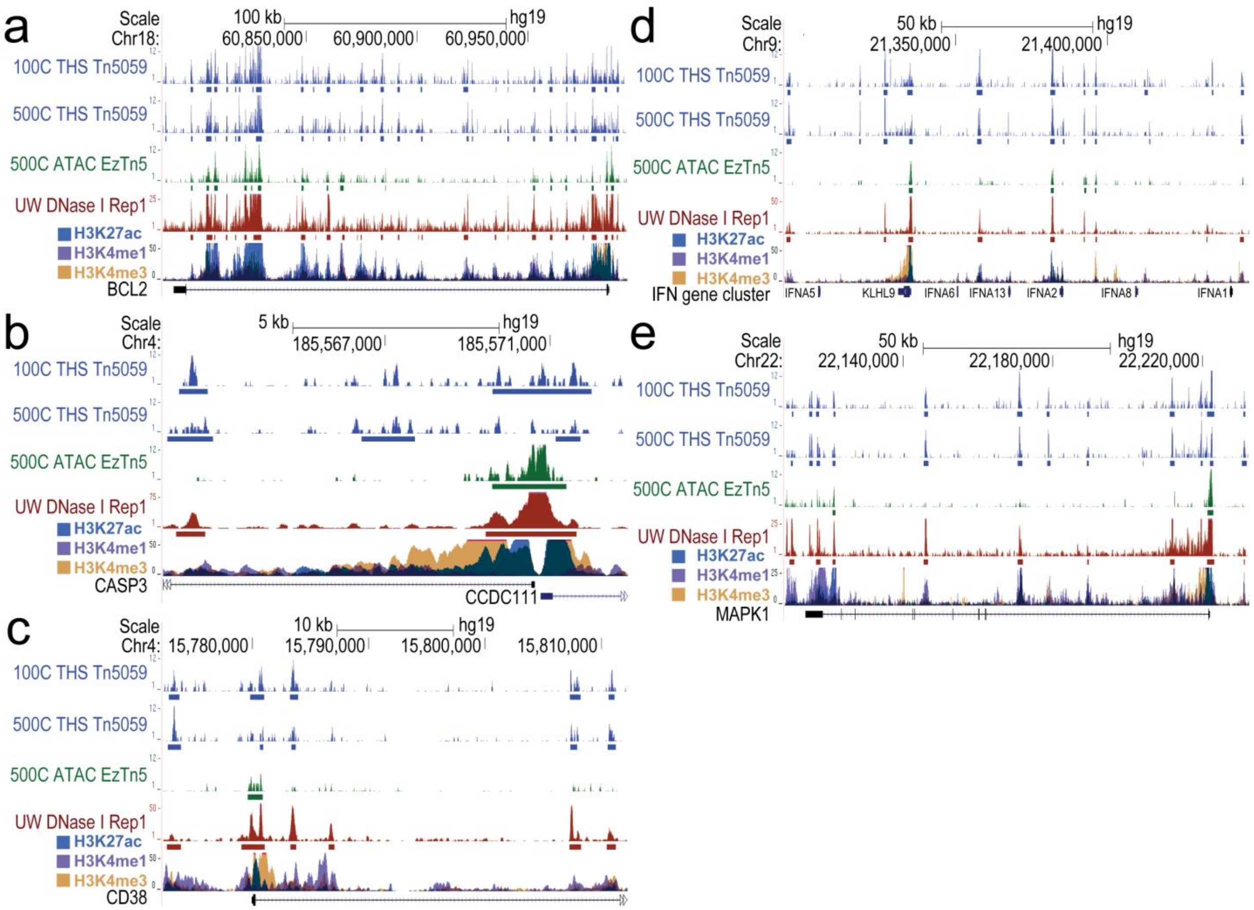

Supp. Figure 17

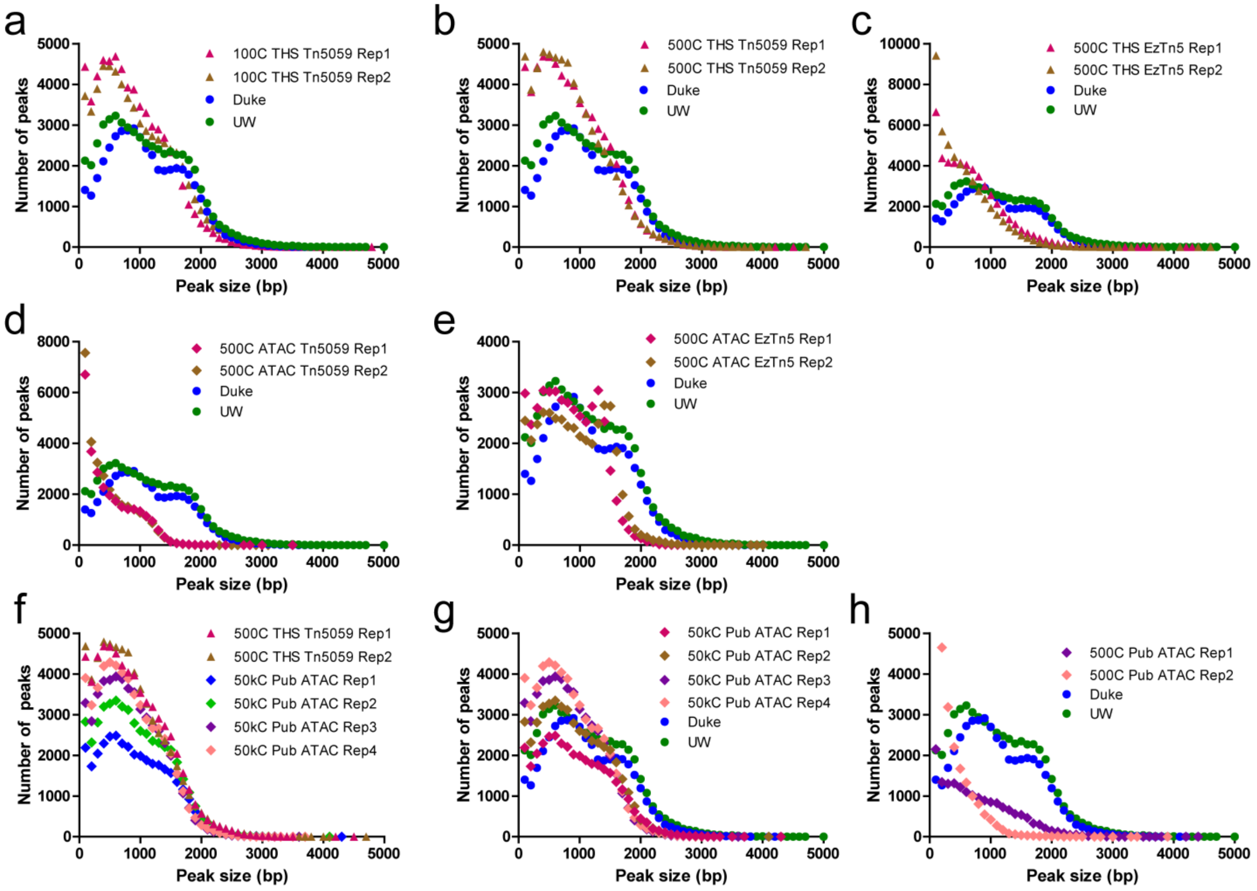

Supp. Figure 18

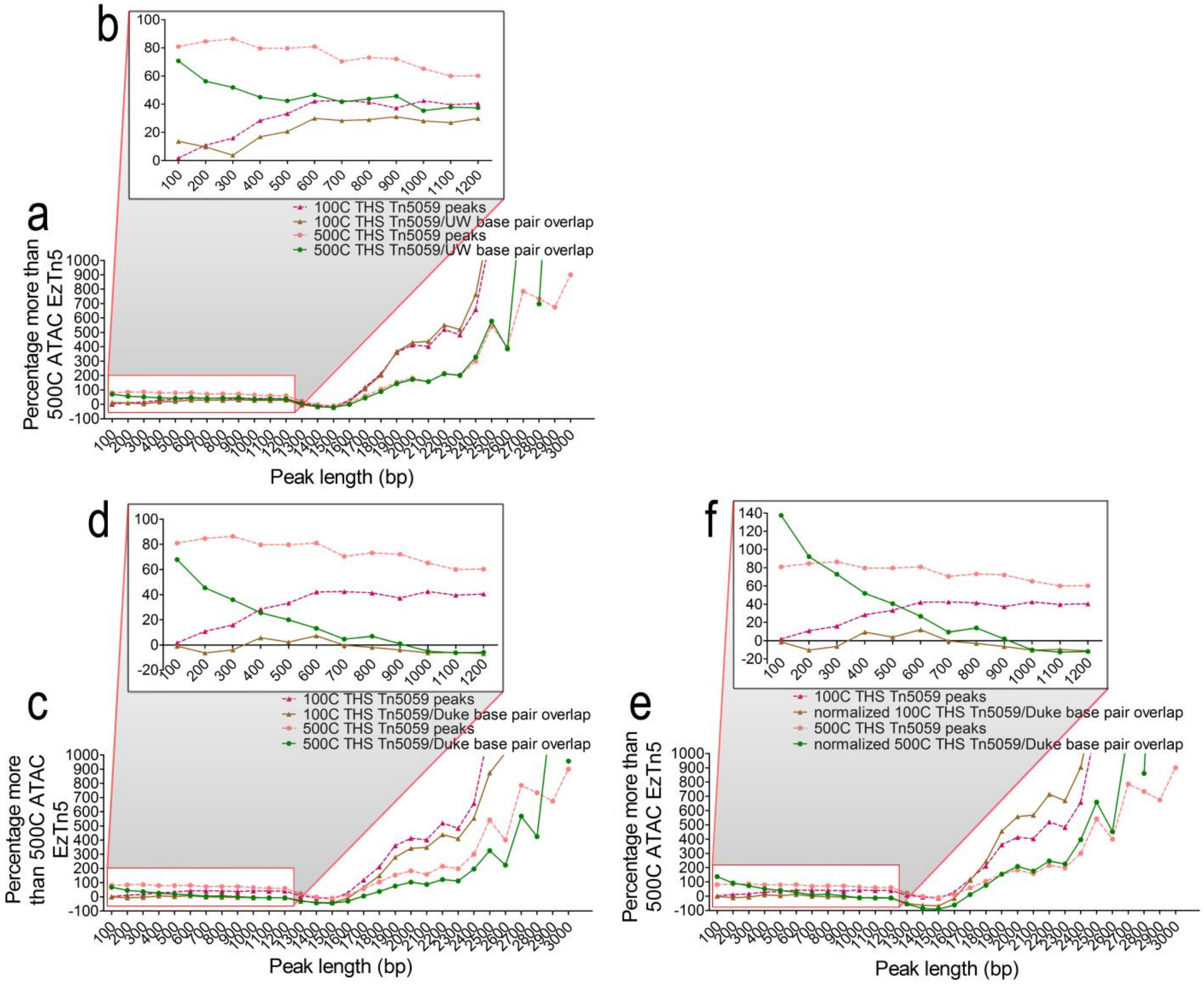

Supp. Figure 19

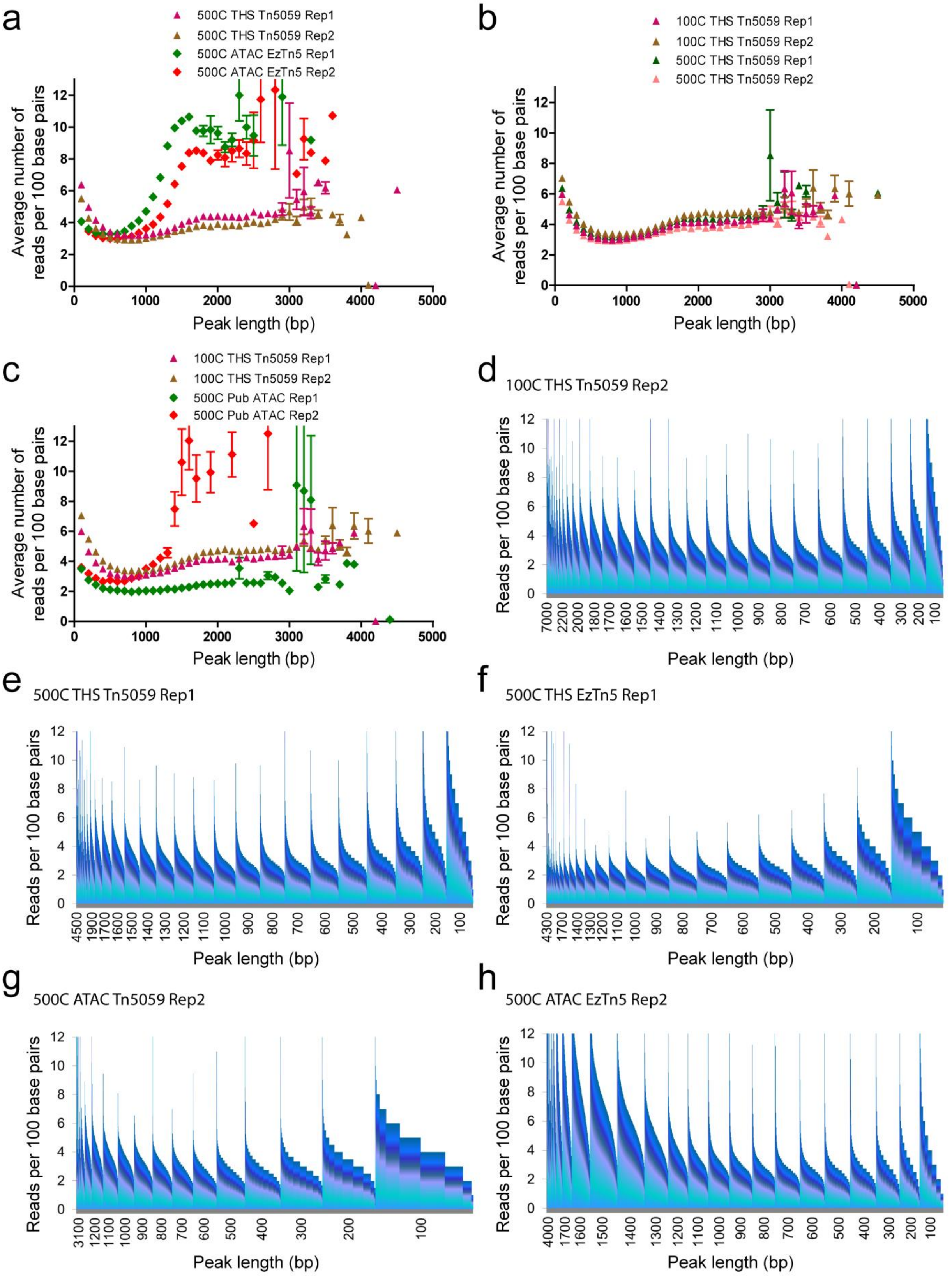

Supp. Figure 20

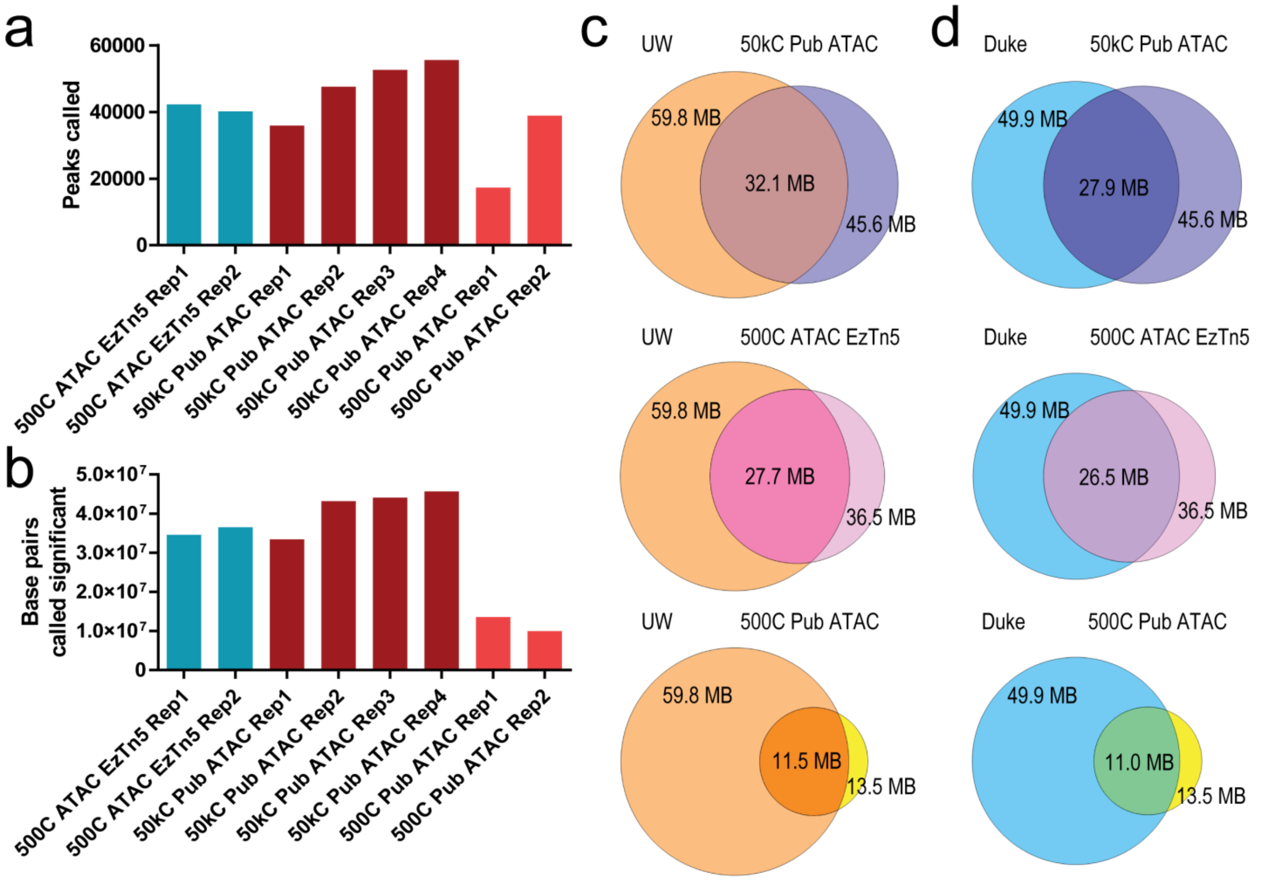

Supp. Figure 21

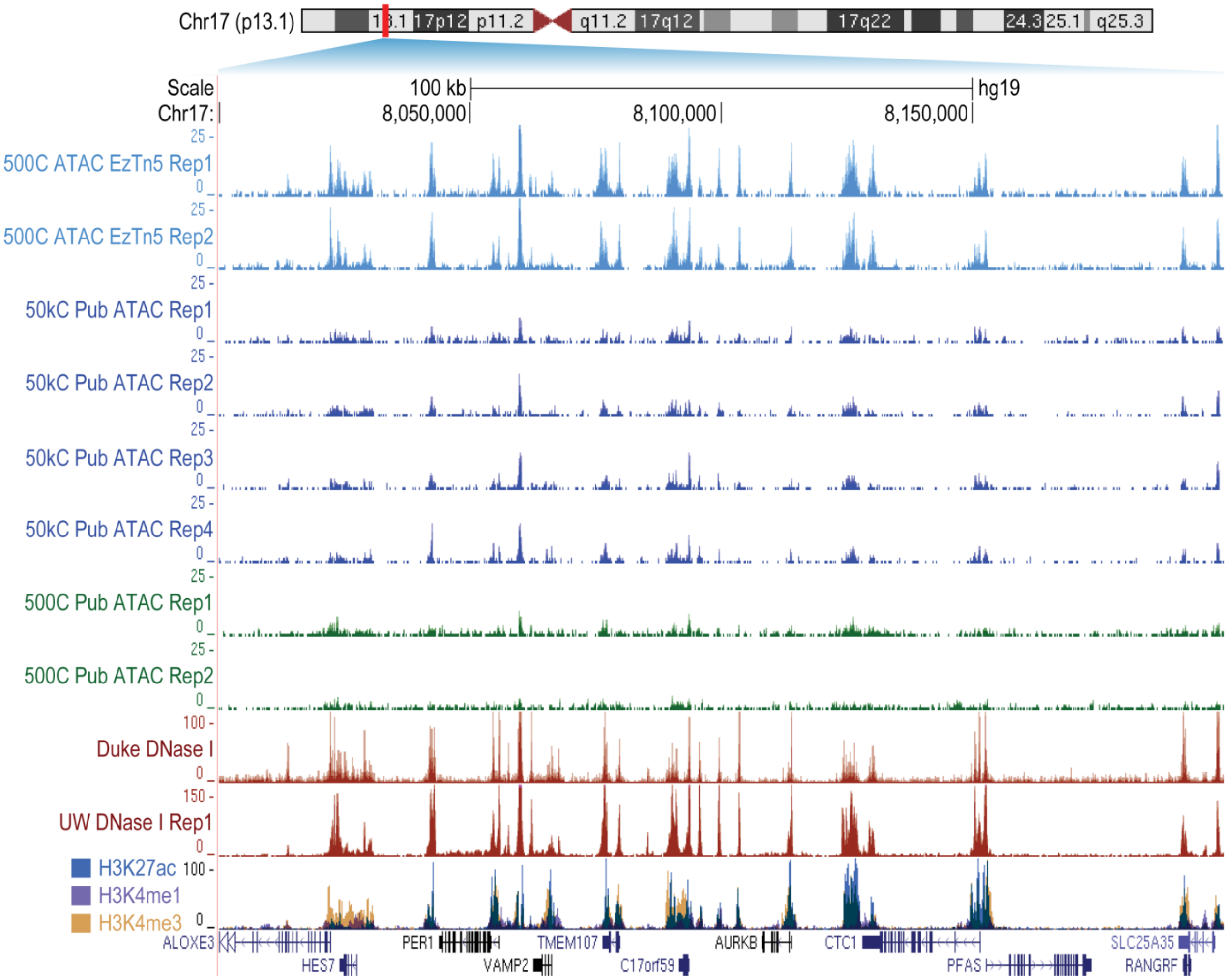

Supp. Table 1

Sample	Total reads	Mapped reads	Mapped %	Clonal reads	Clonal %	Total unique reads	Unique reads %	Peaks
100C_THS_Tn5059_Rep1	83,435,049	77,241,514	92.58%	65,637,161	84.98%	11,604,353	13.91%	63,485
100C_THS_Tn5059_Rep2	71,513,373	65,130,466	91.07%	54,213,284	83.24%	10,917,182	15.27%	62,316
1000C_THS_Tn5059	28,062,259	26,669,972	95.04%	10,598,651	39.74%	16,071,321	57.27%	68,584
NTC_THS_Tn5059	195,832	7,674	3.92%	323	4.21%	7,351	3.75%	-
6_ng_pure_DNA_THS_Tn5059	13,816,259	12,953,563	93.76%	1,098,757	8.48%	11,854,806	85.80%	22,213
Replicate in each sample set with the most number of base pairs called significant (Total coverage) was used for analysis								

Sample	Total coverage	Duke overlap	UW overlap	Duke % overlap	UW % overlap
100C_THS_Tn5059_Rep1	55,039,700	28,235,051	39,333,337	56.59%	65.80%
100C_THS_Tn5059_Rep2	58,602,500	30,593,381	41,951,208	61.32%	70.18%
1000C_THS_Tn5059	57,903,800	24,580,506	36,671,014	49.27%	61.34%
NTC_THS_Tn5059	-	-	-	-	-
6_ng_pure_DNA_THS_Tn5059	3,898,500	76,350	182,964	0.15%	0.31%

SuppTable 2

Peak size (bp)	100C THS Tn5059 Rep1	100C THS Tn5059 Rep2	500C THS Tn5059 Rep1	500C THS Tn5059 Rep2	500C THS EzTn5 Rep1	500C THS EzTn5 Rep2	500C ATAC Tn5059 Rep1	500C ATAC Tn5059 Rep2	500C ATAC EzTn5 Rep1	500C ATAC EzTn5 Rep2	50kC Pub ATAC Rep1	50kC Pub ATAC Rep2	50kC Pub ATAC Rep3	50kC Pub ATAC Rep4
7000	-	-	-	-	-	-	-	-	-	-	-	-	-	-
6900	-	-	-	-	-	-	-	-	-	-	-	-	-	-
6800	-	-	-	-	-	-	-	-	-	-	-	-	-	-
6700	1	1	-	-	-	-	-	-	-	-	-	-	-	-
6300	1	-	-	-	-	-	-	-	-	-	-	-	-	-
6200	-	1	-	-	-	-	-	-	-	-	-	-	-	-
5800	1	1	-	-	-	-	-	-	-	-	-	-	-	-
5500	-	-	-	-	-	-	-	-	-	-	-	-	-	-
5000	-	-	-	-	-	-	-	-	-	-	-	-	-	-
4800	1	1	-	-	-	-	-	-	-	-	-	-	-	-
4700	1	3	-	1	-	-	-	-	-	-	-	-	-	-
4600	1	-	-	-	-	1	-	-	-	-	-	-	-	-
4500	1	-	1	-	-	-	-	-	-	-	-	-	-	-
4400	1	1	-	-	-	1	-	-	-	-	-	-	-	-
4300	-	1	-	-	1	-	-	-	-	-	1	-	-	-
4200	1	-	1	-	1	-	-	-	-	-	-	-	-	-
4100	-	-	-	1	1	1	-	-	-	-	-	1	-	-
4000	-	2	-	1	-	-	-	-	-	1	-	-	-	-
3900	3	1	-	-	-	-	-	-	-	1	-	-	-	-
3800	-	1	-	1	1	2	-	-	-	2	-	-	-	-
3700	-	5	-	3	-	1	-	-	-	-	2	2	-	1
3600	3	2	-	5	1	-	-	-	-	1	-	-	2	-
3500	4	8	2	1	1	-	1	-	-	1	1	1	-	1
3400	3	14	2	9	2	-	-	-	-	2	-	-	-	-
3300	8	19	6	5	1	1	-	-	1	1	3	2	1	2

3200	11	26	8	19	4	-	-	-	2	5	9	7	3	5
3100	13	41	17	22	4	1	-	1	-	1	4	5	1	3
3000	25	64	20	20	10	2	2	1	2	2	11	9	6	4
2900	34	67	31	36	9	2	-	1	4	4	17	16	6	4
2800	55	113	50	48	17	4	1	-	-	6	16	23	10	12
2700	59	147	62	83	25	11	2	1	3	7	31	31	15	17
2600	104	189	105	101	20	12	-	1	4	21	50	51	20	22
2500	130	261	154	155	55	13	-	1	5	24	58	61	30	46
2400	201	331	200	206	60	22	1	-	13	50	112	104	56	68
2300	226	429	253	260	85	38	-	2	19	85	154	145	107	98
2200	347	512	309	322	129	44	2	1	58	98	193	210	122	151
2100	475	678	410	444	173	81	5	2	76	159	346	350	182	200
2000	586	911	569	589	246	93	2	7	135	201	447	411	295	290
1900	814	1177	796	766	333	177	15	12	179	314	617	752	408	469
1800	1044	1529	1161	1028	432	225	33	21	305	568	929	1091	710	694
1700	1510	1980	1567	1372	561	330	51	41	475	991	1165	1419	1069	1117
1600	1956	2327	2045	1741	691	452	79	81	870	1842	1349	1833	1540	1552
1500	2330	2406	2467	2084	832	579	143	151	1464	2734	1567	2136	1992	2043
1400	2691	2556	2722	2359	1126	777	333	304	2433	2753	1661	2208	2304	2408
1300	2894	2639	2908	2547	1367	971	595	545	3045	2384	1766	2310	2604	2664
1200	2964	2728	3194	2859	1710	1268	953	870	2727	1993	1795	2358	2740	2673
1100	3298	2858	3301	3272	2126	1591	1150	1128	2421	2063	1881	2530	2930	2884
1000	3455	3039	3539	3635	2554	1923	1337	1275	2539	2142	1988	2601	3137	3237
900	3875	3428	3968	4036	3008	2369	1417	1405	2666	2305	2023	2795	3552	3500
800	3922	3664	4044	4534	3474	2758	1414	1515	2802	2335	2212	3119	3648	3885
700	4377	4001	4213	4612	3809	3241	1505	1586	2854	2472	2292	3202	3879	4039
600	4684	4315	4508	4661	4036	3734	1731	1836	3025	2491	2486	3350	3940	4211
500	4572	4449	4674	4741	4143	4067	1955	2180	3017	2601	2463	3271	3856	4288
400	4593	4454	4686	4792	4138	4431	2271	2730	3043	2609	2295	3199	3829	4192
300	4197	3891	4431	4396	4159	5032	2861	3242	2697	2377	2042	2809	3509	3666

200	3581	3332	3811	3869	4373	5685	3686	4063	2371	2064	1733	2319	2839	3236
100	4432	3713	4428	4687	6634	9414	6707	7566	2985	2447	2189	2827	3290	3903

Peak size (bp)	500C Pub ATAC Rep1	500C Pub ATAC Rep2	Duke	UW
7000	1	-	-	-
6900	-	-	1	-
6800	-	-	-	1
6700	-	-	-	-
6300	-	-	-	-
6200	-	-	-	-
5800	-	-	-	-
5500	1	-	-	-
5000	-	-	-	2
4800	-	-	-	-
4700	-	-	-	3
4600	-	-	-	1
4500	-	-	-	2
4400	1	-	-	3
4300	-	-	-	1
4200	2	-	1	3
4100	-	-	1	3
4000	-	-	-	10
3900	1	1	-	6
3800	1	-	3	8
3700	1	-	2	9
3600	1	-	5	22
3500	2	-	7	34

3400	1	2	16	22
3300	2	2	15	42
3200	4	-	28	50
3100	4	-	35	75
3000	7	1	60	93
2900	5	-	73	141
2800	5	2	107	164
2700	12	3	136	192
2600	19	-	184	283
2500	31	1	239	344
2400	52	7	299	449
2300	49	7	462	547
2200	88	3	646	746
2100	121	15	870	1078
2000	147	11	1193	1418
1900	201	17	1519	1904
1800	300	13	1782	2140
1700	326	25	1908	2274
1600	466	30	1934	2270
1500	540	41	1902	2346
1400	562	62	1871	2289
1300	661	82	1897	2405
1200	721	170	2259	2477
1100	829	278	2428	2557
1000	854	424	2702	2694
900	893	549	2914	2825
800	959	805	2864	2939
700	1025	999	2851	3062
600	1103	1332	2722	3228
500	1220	1673	2445	3139

400	1315	2200	2106	3012
300	1302	3185	1694	2546
200	1342	4654	1265	2010
100	2154	22304	1401	2121

Supp. Table 3

Sample	Total unique reads	Peaks	Total coverage	Duke overlap	UW overlap	Duke % overlap	UW % overlap	Alignments under peaks
100C_THS_Tn5059_Rep1	8,351,125	58,100	53,753,500	29,577,184	40,382,253	59.28%	67.55%	1,926,239
100C_THS_Tn5059_Rep2	8,351,125	56,514	59,097,200	32,177,819	43,333,154	64.50%	72.49%	2,401,391
500C_THS_Tn5059_Rep1	8,351,125	64,663	56,015,300	24,628,096	35,999,644	49.36%	60.22%	2,083,387
500C_THS_Tn5059_Rep2	8,351,125	64,323	54,271,200	24,134,574	35,015,673	48.38%	58.58%	1,820,897
500C_THS_EzTn5_Rep1	8,351,125	50,352	34,000,500	16,584,237	21,945,810	33.24%	36.71%	905,678
500C_THS_EzTn5_Rep2	8,351,125	49,354	27,420,600	12,733,915	17,279,146	25.52%	28.91%	749,105
500C_ATAC_Tn5059_Rep1	8,351,125	28,252	14,190,500	10,463,017	10,518,599	20.97%	17.60%	541,392
500C_ATAC_Tn5059_Rep2	8,351,125	30,569	14,634,200	10,243,564	10,277,123	20.53%	17.19%	534,833
500C_ATAC_EzTn5_Rep1	8,351,125	42,240	34,575,500	26,615,551	27,439,757	53.35%	45.90%	2,254,535
500C_ATAC_EzTn5_Rep2	8,351,125	40,157	36,477,100	26,543,578	27,668,343	53.20%	46.28%	1,998,408
50kC_Pub_ATAC_Rep1	8,351,125	35,908	33,400,400	24,772,383	27,067,883	49.65%	45.28%	-
50kC_Pub_ATAC_Rep2	8,351,125	47,558	43,184,100	28,993,599	32,932,251	58.12%	55.09%	-
50kC_Pub_ATAC_Rep3	8,351,125	52,632	44,060,100	27,172,983	30,810,815	54.47%	51.54%	-
50kC_Pub_ATAC_Rep4	8,351,125	55,585	45,643,500	27,849,765	32,074,371	55.82%	53.66%	-
500C_Pub_ATAC_Rep1	8,351,125	17,331	13,540,000	11,003,510	11,456,205	22.06%	19.16%	-
500C_Pub_ATAC_Rep2	7,538,481	38,898	9,934,200	4,410,337	4,769,889	8.84%	7.98%	-
Replicate in each sample set with the most number of base pairs called significant (Total coverage) was used for analysis								

Supp. Table 4

Sample	Total Reads	Mapped Reads	Mapped %	Clonal Reads	Clonal %	Total unique reads	Peaks	Total coverage
100C_THS_Tn5059_Rep1	83,435,049	77,241,514	92.58%	65,637,161	84.98%	11,604,353	63,485	55,039,700
100C_THS_Tn5059_Rep2	71,513,373	65,130,466	91.07%	54,213,284	83.24%	10,917,182	62,316	58,602,500
500C_THS_Tn5059_Rep1	36,656,474	33,222,486	90.63%	15,142,913	45.58%	18,079,573	65,109	59,052,900
500C_THS_Tn5059_Rep2	43,652,122	39,820,044	91.22%	18,801,045	47.22%	21,018,999	61,678	59,997,600
500C_THS_EzTn5_Rep1	42,374,624	39,142,475	92.37%	7,682,430	19.63%	31,460,045	57,656	49,323,000
500C_THS_EzTn5_Rep2	49,786,595	46,097,848	92.59%	9,121,951	19.79%	36,975,897	52,544	47,328,300
500C_ATAC_Tn5059_Rep1	19,760,628	19,370,405	98.03%	5,111,053	26.39%	14,259,352	31,967	13,024,200
500C_ATAC_Tn5059_Rep2	24,827,669	24,300,992	97.88%	7,603,292	31.29%	16,697,700	30,810	13,916,800
500C_ATAC_EzTn5_Rep1	23,336,416	21,400,014	91.70%	11,570,533	54.07%	9,829,481	43,630	36,711,000
500C_ATAC_EzTn5_Rep2	18,624,957	16,319,759	87.62%	7,968,634	48.83%	8,351,125	40,157	36,477,100
50kC_Pub_ATAC_Rep1	192,904,649	189,145,446	98.05%	146,210,593	77.30%	42,934,853	48,869	50,185,100
50kC_Pub_ATAC_Rep2	56,598,621	55,704,080	98.42%	42,835,039	76.90%	12,869,041	50,340	47,462,300
50kC_Pub_ATAC_Rep3	85,243,035	84,161,992	98.73%	70,278,832	83.50%	13,883,160	53,501	50,384,900
50kC_Pub_ATAC_Rep4	62,821,955	61,950,510	98.61%	51,169,895	82.60%	10,780,615	58,385	48,154,500
500C_Pub_ATAC_Rep1	23,259,350	22,517,253	96.81%	11,290,578	50.14%	11,226,675	27,494	12,828,800
500C_Pub_ATAC_Rep2	22,898,819	22,403,334	97.84%	14,864,853	66.35%	7,538,481	38,898	9,934,200

Replicate in each sample set with the most number of base pairs called significant (Total coverage) was used for analysis

Sample	Duke overlap	UW overlap	Duke % overlap	UW % overlap
100C_THS_Tn5059_Rep1	28,235,051	39,333,337	56.59%	65.80%
100C_THS_Tn5059_Rep2	30,593,381	41,951,208	61.32%	70.18%
500C_THS_Tn5059_Rep1	24,797,108	37,174,108	49.70%	62.19%
500C_THS_Tn5059_Rep2	25,018,762	37,460,275	50.15%	62.66%
500C_THS_EzTn5_Rep1	21,427,026	29,659,156	42.95%	49.61%
500C_THS_EzTn5_Rep2	19,133,219	27,231,310	38.35%	45.55%
500C_ATAC_Tn5059_Rep1	8,365,696	8,676,219	16.77%	14.51%
500C_ATAC_Tn5059_Rep2	8,870,056	9,182,873	17.78%	15.36%
500C_ATAC_EzTn5_Rep1	26,391,981	27,437,896	52.90%	45.90%
500C_ATAC_EzTn5_Rep2	26,543,578	27,668,343	53.20%	46.28%
50kC_Pub_ATAC_Rep1	31,243,373	36,079,092	62.62%	60.35%
50kC_Pub_ATAC_Rep2	29,503,465	34,317,340	59.14%	57.41%
50kC_Pub_ATAC_Rep3	28,631,225	33,293,399	57.39%	55.69%
50kC_Pub_ATAC_Rep4	27,640,100	32,431,186	55.40%	54.25%
500C_Pub_ATAC_Rep1	7,961,231	8,555,233	15.96%	14.31%
500C_Pub_ATAC_Rep2	4,410,337	4,769,889	8.84%	7.98%

Supp. Table 5

Name	DNA sequence	notes
T7tspn-top2	5'- CATGAGATTAATACGACTCACTATAGGGAGATCCT CCCTCGCGCCATCAGAGATGTGTATAAGAGACAG -3'	PAGE purification
T7tspn-bot	5'- /5Phos/CTGTCTCTTATACACATCT -3'	5Phos
sss_NPA_prmr	5'- /5Phos/GGGAGATCCTCCCTCGCGCCATCAGAGATGTGTATAAGAGACAG -3'	5Phos
ME_BOT	5'- /5Phos/CTGTCTCTTATACACATCT -3'	5Phos
ME_TOP	5'- GCCTTGCCAGCCCGCTCAGAGATGTGTATAAGAGACAG -3'	-
PCR_5'_primer	5'- AATGATACGGCGACCACCGAAGATCCTCCCTCGCGCCATCAGAG -3'	-
Nxta_Ind49	5'- CAAGCAGAAGACGGCATACGAGATACACAGCGGTCTGCCTTGCCAGCCCGCTCAG -3'	-
Nxta_Ind50	5'- CAAGCAGAAGACGGCATACGAGATAAAGGTCGGTCTGCCTTGCCAGCCCGCTCAG -3'	-
Nxta_Ind51	5'- CAAGCAGAAGACGGCATACGAGATGCGATACGGTCTGCCTTGCCAGCCCGCTCAG -3'	-
Nxta_Ind52	5'- CAAGCAGAAGACGGCATACGAGATCGTGTCCGGTCTGCCTTGCCAGCCCGCTCAG -3'	-
Nxta_Ind53	5'- CAAGCAGAAGACGGCATACGAGATGTAGAACGGTCTGCCTTGCCAGCCCGCTCAG -3'	-
Nxta_Ind54	5'- CAAGCAGAAGACGGCATACGAGATGGACGTCGGTCTGCCTTGCCAGCCCGCTCAG -3'	-
Nxta_Ind55	5'- CAAGCAGAAGACGGCATACGAGATAGTCGACGGTCTGCCTTGCCAGCCCGCTCAG -3'	-
Nxta_Ind56	5'- CAAGCAGAAGACGGCATACGAGATGTCTGACGGTCTGCCTTGCCAGCCCGCTCAG -3'	-
Nxta_Ind57	5'- CAAGCAGAAGACGGCATACGAGATGAAGGACGGTCTGCCTTGCCAGCCCGCTCAG -3'	-
Nxta_Ind58	5'- CAAGCAGAAGACGGCATACGAGATATGCTGCGGTCTGCCTTGCCAGCCCGCTCAG -3'	-
Nxta_Ind59	5'- CAAGCAGAAGACGGCATACGAGATTCTATCCGGTCTGCCTTGCCAGCCCGCTCAG -3'	-
Nxta_Ind60	5'- CAAGCAGAAGACGGCATACGAGATATCTGTCGGTCTGCCTTGCCAGCCCGCTCAG -3'	-
Nxta_Ind61	5'- CAAGCAGAAGACGGCATACGAGATATAGAGCGGTCTGCCTTGCCAGCCCGCTCAG -3'	-
Nxta_Ind62	5'- CAAGCAGAAGACGGCATACGAGATGCTAAACGGTCTGCCTTGCCAGCCCGCTCAG -3'	-
Nxta_Ind63	5'- CAAGCAGAAGACGGCATACGAGATACCAGGCGGTCTGCCTTGCCAGCCCGCTCAG -3'	-
Nxta_Ind64	5'- CAAGCAGAAGACGGCATACGAGATCCAACTCGGTCTGCCTTGCCAGCCCGCTCAG -3'	-
Nxta_Ind65	5'- CAAGCAGAAGACGGCATACGAGATAAGGAACGGTCTGCCTTGCCAGCCCGCTCAG -3'	-
Nxta_Ind66	5'- CAAGCAGAAGACGGCATACGAGATCCTCCACGGTCTGCCTTGCCAGCCCGCTCAG -3'	-
Nxta_Ind67	5'- CAAGCAGAAGACGGCATACGAGATCACGTCCGGTCTGCCTTGCCAGCCCGCTCAG -3'	-
Nxta_Ind68	5'- CAAGCAGAAGACGGCATACGAGATCATAACCGGTCTGCCTTGCCAGCCCGCTCAG -3'	-
Nxta_Ind69	5'- CAAGCAGAAGACGGCATACGAGATCCATATCGGTCTGCCTTGCCAGCCCGCTCAG -3'	-
Nxta_Ind70	5'- CAAGCAGAAGACGGCATACGAGATGAAGTCCGGTCTGCCTTGCCAGCCCGCTCAG -3'	-

Nxta_Ind71	5'- CAAGCAGAAGACGGCATACGAGATCAAAGACGGTCTGCCTTGCCAGCCCGCTCAG -3'	-
Nxta_Ind72	5'- CAAGCAGAAGACGGCATACGAGATTGGCAGCGGTCTGCCTTGCCAGCCCGCTCAG -3'	-
Nxta_seq_Indx_read	5'- CTGTCTCTTATACACATCTCTGAGCGGGCTGGCAAGGCAGACCG -3'	-
Nxta_seq_Read1_long	5'- CCGAAGATCCTCCCTCGCGCCATCAGAGATGTGTATAAGAGACAG -3'	-
